# Macromolecular assembly of bioluminescent protein nanoparticles for enhanced imaging

**DOI:** 10.1016/j.mtbio.2022.100455

**Published:** 2022-10-08

**Authors:** Enya Li, Caroline K. Brennan, Aaron Ramirez, Jo A. Tucker, Nina Butkovich, Vijaykumar S. Meli, Anastasia A. Ionkina, Edward L. Nelson, Jennifer A. Prescher, Szu-Wen Wang

**Affiliations:** a Department of Chemical & Biomolecular Engineering, University of California, Irvine, CA, 92697, USA; b Department of Chemistry, University of California, Irvine, CA, 92697, USA; c Department of Medicine, University of California, Irvine, CA, 92697, USA; d Department of Biomedical Engineering, University of California, Irvine, CA, 92697, USA; e Department of Molecular Biology & Biochemistry, University of California, Irvine, CA, 92697, USA; f Chao Family Comprehensive Cancer Center, University of California, Irvine, CA, 92697, USA; g Department of Pharmaceutical Sciences, University of California, Irvine, CA, 92697, USA

**Keywords:** Nanoparticle, Bioluminescence, NanoLuc luciferase, Akaluc luciferase, *In vitro* imaging, *In vivo* imaging

## Abstract

Bioluminescence imaging has advantages over fluorescence imaging, such as minimal photobleaching and autofluorescence, and greater signal-to-noise ratios in many complex environments. Although significant achievements have been made in luciferase engineering for generating bright and stable reporters, the full capability of luciferases for nanoparticle tracking has not been comprehensively examined. In biocatalysis, enhanced enzyme performance after immobilization on nanoparticles has been reported. Thus, we hypothesized that by assembling luciferases onto a nanoparticle, the resulting complex could lead to substantially improved imaging properties. Using a modular bioconjugation strategy, we attached NanoLuc (NLuc) or Akaluc bioluminescent proteins to a protein nanoparticle platform (E2), yielding nanoparticles NLuc-E2 and Akaluc-E2, both with diameters of ∼45 ​nm. Although no significant differences were observed between different conditions involving Akaluc and Akaluc-E2, free NLuc at pH 5.0 showed significantly lower emission values than free NLuc at pH 7.4. Interestingly, NLuc immobilization on E2 nanoparticles (NLuc-E2) emitted increased luminescence at pH 7.4, and at pH 5.0 showed over two orders of magnitude (>200-fold) higher luminescence (than free NLuc), expanding the potential for imaging detection using the nanoparticle even upon endocytic uptake. After uptake by macrophages, the resulting luminescence with NLuc-E2 nanoparticles was up to 7-fold higher than with free NLuc at 48 ​h. Cells incubated with NLuc-E2 could also be imaged using live bioluminescence microscopy. Finally, biodistribution of nanoparticles into lymph nodes was detected through imaging using NLuc-E2, but not with conventionally-labeled fluorescent E2. Our data demonstrate that NLuc-bound nanoparticles have advantageous properties that can be utilized in applications ranging from single-cell imaging to *in vivo* biodistribution.

## Graphical abstract

**Figure:**
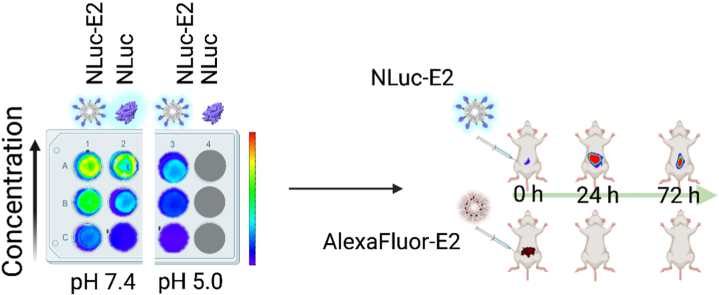

## 1 Introduction

Optical imaging techniques, including fluorescence and bioluminescence, have been widely used for visualizing biological features in cells and *in vivo* [[1], [2], [3]]. Unlike fluorescence, bioluminescence does not require excitation light. Instead, the luciferase enzyme oxidizes its substrate (luciferin), generating an excited-state oxyluciferin product; light is generated when the oxyluciferin returns to ground state [3]. Fluorescent molecules are susceptible to photobleaching and autofluorescence, while bioluminescent probes provide low background and high signal-to-noise ratios [1]. However, suboptimal resolution is often encountered in both fluorescence and bioluminescence imaging due to light absorption and photon scattering [2,4]. Recent advancements have been made in the development of brighter bioluminescent reporters [5]. However, the full potential of luciferases on nanoparticles for improved imaging has not been extensively studied.

Our research team uses the E2 protein nanoparticle platform for drug delivery and cancer vaccine development [[6], [7], [8], [9]]. E2 is a 60-mer protein derived from the pyruvate dehydrogenase complex of *Geobacillus stearothermophilus* and is approximately 25-nm in diameter with a 12-nm internal cavity that forms a dodecahedral caged structure [10]. E2 has been shown to have exceptional thermostability, and can be engineered at the internal, external, and inter-subunit surfaces [7,10,11]. In the general area of biocatalysis, investigations have shown that immobilizing enzymes onto nanoparticles can increase their enzymatic activity, thermal stability, and half-life [12,13]. Thus, we hypothesized that by immobilizing luciferases onto the exterior surface of the E2 nanoparticle, we would observe enhanced luciferase performance.

We chose NanoLuc (NLuc), Akaluc, and Alexa Fluor 750 (AF) as the imaging molecules to attach to E2. NLuc is a 19 ​kDa luciferase from the deep-sea shrimp, *Oplophorus gracilirostris*, that emits blue light (λ_max_^EM^ ​= ​460 ​nm) [14]. It is small relative to the commonly used firefly and *Renilla* luciferases, and is ATP-independent [14,15]. Furthermore, NLuc paired with its substrate furimazine, an optimized coelenterazine derivative, is approximately 150-fold brighter than firefly and *Renilla* luciferases [14,15]. Many recent efforts have focused on developing red-shifted bioluminescent reporters for *in vivo* imaging [16]. Included in this group is Akaluc, a 61 ​kDa luciferase with 28 amino acid substitutions compared to firefly luciferase. Akaluc exhibits maximum photon emission at 650 ​nm [17], with sufficient tissue-penetrant light for imaging in deep tissues, including in the lungs and striatum [17]. Alexa Fluor 750 was chosen as the fluorescent molecule for comparison since its emission wavelength is in the near-infrared window, which can lead to attenuated photon absorbance, scattering, and autofluorescence, and greater penetration depth [18]. Recombinantly fusing proteins (e.g., NLuc, Akaluc) to self-assembling nanoparticles such as E2 can result in expression and folding difficulties; thus, we implemented the SpyCatcher-SpyTag strategy to attach NLuc and Akaluc to E2 [19,20]. This system enables us to express and purify each protein component separately, then conjugate them together. The lysine on SpyCatcher (SC) and the aspartic acid on SpyTag (ST) form a covalent isopeptide bond that is robust under a range of pH, temperature, and buffer conditions [19,20]. The SC-ST strategy has been utilized to decorate various virus-like particles, including bacteriophage AP205 [[21], [22], [23]], hepatitis B [24], ferritin [25], E2 [26,27], and others [20,28].

For this study, we constructed NLuc and Akaluc attached to the E2 nanoparticle (NLuc-E2, Akaluc-E2). The size of the bioluminescent E2 constructs is optimal for uptake by antigen-presenting cells (APCs) [[29], [30], [31]]. Uptake of nanoparticles is often followed by encounters with the early endosome, late endosome, and lysosome [32], which range in pH from 4.5 to 6.5 [33,34]. Thus, we aimed to understand whether NLuc-E2 and Akaluc-E2 can still maintain their enzymatic activity under both typical physiological (pH 7.4) and acidic (pH 5.0) pH conditions. We also examined their uptake by antigen-presenting cells and investigated the persistence of luminescence in cells over time. Finally, we used NLuc-E2 for live cell, *in vivo*, and *ex vivo* imaging, and compared these results with fluorescently-labeled nanoparticles.

## 2 Materials and methods

### 2.1 Materials

Reagents were purchased from Fisher Scientific unless mentioned otherwise. All restriction enzymes and ligase were purchased from New England Biolabs (NEB). General cloning and protein expression were performed using DH5α and BL21(DE3) cell lines, respectively. We used QIAprep Spin Miniprep Kits (QIAGEN) for minipreps and GeneJET Gel Extraction Kits for agarose gel extraction. The DNA oligonucleotides for cloning were purchased from Integrated DNA Technologies (Coralville, IA). Polymerase chain reactions (PCRs) were performed using a CloneJET PCR cloning kit. The plasmid containing the SpyCatcher gene (pDEST14-SpyCatcher) was from Addgene. The primer sequences for PCR and the amino acid sequences for the final proteins expressed can be found in the Supporting Information (Table SI-1).

### 2.2 ST-E2 cloning, expression, and purification

All E2 protein nanoparticles used in this study were derived from the previously-described D381C mutant [10], which contains 60 cysteines in the internal hollow cavity for functionalization, and is abbreviated “E2”. SpyTag (ST) was recombinantly attached to each E2 (D381C) monomer via an 11-amino acid long linker with a protein sequence of GSGTAGGGSGS to yield ST-E2. This was performed via PCR using the primers *Nde*I-SpyTag_Forward and *Bam*HI-SpyTag_Reverse primers: 5′-CATATGGCCCACATCGTTATGGTGGATGCCTACAAGCCAACTAAAGGTTCAGGAACAGCAGGTGGTGGGTCAGGTTCCCTGTCTGTTCCTGGTCCCGC -3′ ​and ​5′-GGATCCTTAAGCTTCCATCAGC.

AGCAGTTCCGG-3’, respectively. A standard PCR protocol was used, with the enzyme Phusion High-Fidelity DNA polymerase and the D381C gene as the DNA template. The subsequent PCR-synthesized ST-E2 gene was then inserted into pJET1.2/blunt vector (CloneJet PCR cloning kit) and the gene sequence was confirmed (Azenta). This gene was then cloned into *Nde*I and *Bam*HI sites on a pET11a vector expression vector using T4 DNA ligase. The pET11a-ST-E2 plasmid was transformed into *E. coli* strain BL21(DE3).

The expression and purification steps of ST-E2 were performed similarly to previously described mutants [10,35]. In brief, pET11a-ST-E2 in BL21(DE3) was inoculated in Luria-Bertani medium with 100 ​μg/mL of ampicillin and cultured at 37 ​°C until an OD_600_ of 0.6–0.9 was reached. IPTG (1 ​mM) was added, and cells were cultured for 3 ​h. Cells were centrifuged, pelleted, and stored at −80 ​°C overnight. To lyse, cells were resuspended in breaking buffer (20 ​mM Tris pH 8.7, 0.02% sodium azide, 2 U/mL DNase, 2 U/mL RNase, 1 ​mM MgCl_2_, and 1 ​mM PMSF), and lysed with French Press (Thermo). ST-E2 was purified by removing insoluble proteins at 70 ​°C by ultracentrifugation at 100,000×*g*, followed by FPLC purification using HiPrep Q Sepharose anion exchange (GE Healthcare) and Superose 6 size exclusion (GE Healthcare) columns.

### 2.3 Cloning, purification, and characterization of the bioluminescent proteins

To obtain SpyCatcher (SC)-bioluminescent proteins, SpyCatcher (SC) with a 6x-His tag on its N-terminus was PCR amplified and cloned into the pET11a vector using *Nde*I and *Nhe*I sites. These cut sites on the N- and C-termini of SC were introduced using the *Nde*I-SpyCatcher_Forward and *Nhe*I-SpyCatcher_Reverse primers: 5′- CATATGTCGTACTACCATCACCATCACCATCACG-3′ and 5′- GCTAGCAATATGAGCGTCACCTTTAGTTGCTTTGCC -3′, respectively. To insert the bioluminescent proteins, *Nhe*I and *Bam*HI restriction sites were added through PCR to the N- and C-termini of NLuc and Akaluc (both plasmids described previously [36]) with primers *Nhe*I-NLuc_Forward, *Bam*HI-NLuc_Reverse, *Nhe*I-Akaluc_Forward, and *Bam*HI-Akaluc_Reverse. The forward primer sequence included the same 11 amino acid spacer used for ST-E2 construction at the N-terminus of NLuc or Akaluc. NLuc or Akaluc were ligated with SpyCatcher via *Nhe*I and *Bam*HI cut sites resulting in the SpyCatcher-bioluminescent fusion protein. The plasmid was transformed into BL21(DE3) for protein expression and purification.

Cells containing the SC-NLuc or SC-Akaluc gene were inoculated in Luria-Bertani medium with 100 ​μg/mL of ampicillin at 37 ​°C and induced using 1 ​mM of IPTG at an OD_600_ between 0.6 and 0.9. SC-NLuc was expressed at 37 ​°C for 3 ​h, and SC-Akaluc was expressed at 20 ​°C overnight. Cells were pelleted and frozen at −80 ​°C overnight. Cells were lysed with French press and the soluble cell lysate was separated from the insoluble fraction after ultracentrifugation. Purification of SC-fusion proteins were performed using HisPur™ Ni-NTA spin columns (Thermo Scientific) following the manufacturer's protocol. Collected fractions were analyzed using SDS-PAGE. Fractions containing the purified protein of interest were pooled together, and the purified proteins were dialyzed in phosphate-buffered saline (PBS) to remove imidazole.

NLuc without SpyCatcher was inserted into the pET11a vector through PCR using *Nde*I and *Bam*HI cut sites. *Nde*I and ​*Bam*HI cut sites were introduced into NLuc via the primers *Nde*I-NLuc 6x HIS_Forward and *Bam*HI-NLuc 6x HIS_Reverse (Supporting Information, Table 1). In addition, a 6x-His tag was introduced to the C-terminus of NLuc. Akaluc without SpyCatcher in pET28a contained a 6x-His tag on the N-terminus. Both NLuc and Akaluc genes were transformed into BL21(DE3) for protein expression. NLuc or Akaluc were expressed in Luria-Bertani medium with 100 ​μg/mL of ampicillin or 50 ​μg/mL of kanamycin, respectively. The same expression and purification steps discussed above for SC-NLuc or SC-Akaluc were followed for expressing and purifying NLuc or Akaluc.

The molecular weight and purity for all proteins were determined with SDS-PAGE, and the concentration was measured with a BCA protein assay (Pierce). Lipopolysaccharide from all proteins for *in vitro* and *in vivo* work were removed with Triton X-114 (Sigma), and the endotoxin levels were confirmed to be lower than 0.1 EU/mg, as evaluated by LAL ToxinSensor (Genscript).

To predict the isoelectric point (pI) of ST-E2, SC-NLuc and NLuc, the protein sequences of each protein were entered into the EMBL-EBI calculator [37]. ChimeraX was utilized to verify the location of the active site of NLuc, using structural information from the Protein Data Bank (ID code 5IBO) [38,39].

### 2.4 Conjugation of imaging molecules to E2

SpyCatcher-bioluminescent proteins were incubated with ST-E2 particles at a range of molar ratios from 0.6:1 to 1:1 (SC-bioluminescent protein:ST-E2 monomer). The molar ratio resulting in the maximized number of immobilized bioluminescent protein on E2 while also minimizing the amount of unconjugated bioluminescent protein was selected as the molar ratio of choice. To synthesize E2 particles loaded with bioluminescent protein for *in vitro* and *in vivo* imaging assays, molar ratios of 0.7:1 and 0.6:1, for SC-NLuc:ST-E2 and SC-Akaluc:ST-E2, respectively, were used. The proteins were mixed in phosphate buffer (50 ​mM potassium phosphate and 100 ​mM NaCl at pH 7.4) and allowed to react at room temperature for 2 ​h, followed by 4 ​°C overnight to obtain the bioluminescent protein nanoparticles (NLuc-E2 or Akaluc-E2). The conjugation ratio of bioluminescent protein to E2 nanoparticle was evaluated based on densitometry of the SDS-PAGE gels measured with ImageJ [40]. The size and monodispersity of the particles and ST-E2 were measured with dynamic light scattering (Malvern Zetasizer, Nano ZS) and the concentration was determined with BCA protein assay. Alexa Fluor 750 C_5_-maleimide was conjugated to the internal cysteines of E2(D381C) as previously described [10], and details are given in Supporting Information.

Nanoparticles were also imaged with transmission electron microscopy (TEM). NLuc-E2 was conjugated with aldehyde-terminated CpG-1826 to the interior cavity of E2 to aid with negative staining [8]. The nanoparticles (0.04 ​mg/mL) were stained with 2% uranyl acetate on carbon-coated copper grids (Ted Pella) and imaged with a JEM-2100 ​F transmission electron microscope operating at 200 ​kV.

### 2.5 *In vitro* luminescence from the NLuc and akaluc constructs at different pH conditions

Luminescence of NLuc or Akaluc samples were loaded into black 96-well plates with clear flat bottoms (Corning) and measured using an IVIS Lumina CCD camera (PerkinElmer, UK) after furimazine or MgSO_4_, ATP, and Akalumine-HCl addition. The CCD camera was chilled to −90 ​°C and the imaging stage was maintained at 37 ​°C for the duration of imaging. The excitation and emission filters used block and open settings, respectively. Exposure time ranging from 0.5 to 2 ​s, binning levels of medium, field of view of 12.5 ​cm, and f number of 1 were set. NLuc and Akaluc samples were serially diluted in phosphate buffer (50 ​mM potassium phosphate and 100 ​mM NaCl) with a pH of 7.4 or 5.0 for the NLuc and Akaluc samples. A 0.1 ​M citrate buffer (58 ​mM sodium citrate dihydrate and 42 ​mM citric acid) with a pH of 5.0 was also utilized as a dilution buffer since its buffering capacity was greater. Before imaging, 1:50 dilution of furimazine in the imaging buffer (Nano-Glo luciferase assay system kit, Promega, UK) was added to samples containing NLuc and to the buffer controls. 5 ​mM MgSO_4_, 80 ​μM ATP and 150 ​μM Akalumine-HCl (TokeOni, Sigma-Aldrich) were added to samples with Akaluc and the buffer controls. Living Image software was utilized to select regions of interest after imaging, and total flux was quantified.

### 2.6 *In vitro* luminescence from the NLuc constructs taken up by macrophages

RAW 264.7 ​cells (ATCC) were seeded in a 96-well clear bottom white plate at a density of 10,000 ​cells per well and allowed to settle for 20–24 ​h. The cells were cultured in macrophage media [DMEM (Gibco) with 10% FBS (Life Technologies)] and in an incubator at 37 ​°C with 5% CO_2_. Per well, 100 ​μL of media containing 100 ​nM or 30 ​nM NLuc (on NLuc-E2 nanoparticles or free in solution) was added to cells for incubation.

Cells were incubated with these NLuc-E2 or free NLuc conditions for an hour at 37 ​°C. After this incubation period, residual NLuc-E2 or NLuc were removed by washing with media three times, followed by an additional incubation period of 0, 3, 24 or 48 ​h at 37 ​°C (defined as the “time inside cells”) to examine the persistence of the bioluminescent signal within cells. Before measuring luminescence, 50 ​μL of 1:50 dilution of furimazine (Promega) in media was added to each well containing 50 ​μL of fresh media and allowed to incubate for at least 3 ​min at room temperature. Luminescence was measured with a plate reader (SpectraMax M3). Because furimazine can be toxic to cells [41], the cells measured at different timepoints were not from the same wells. Instead, multiple wells with cells were incubated for the different indicated times and the luminescence was measured only at the assigned timepoint for each well. Luminescence is measured in relative luminescence units (RLU) and plotted with the background reading from media-only controls subtracted.

### 2.7 Bioluminescent microscopy of NLuc-E2 or NLuc uptake by APCs

NLuc-E2 was visualized using a specially developed bioluminescence microscope [42]. Samples were placed in a temperature-, humidity-, and CO_2_-controlled box (Tokai) within an Olympus IX83 TIRF microscope, which was used in widefield mode. The signal was collected with a sCMOS camera (Prime 95 ​b, Photometrics, operated in cooled mode). The acquisition was controlled by the μManager software. Mouse bone marrow-derived dendritic cells (BMDCs, differentiated as we have previously described [43]) or RAW 264.7 macrophage cells (100,000 ​cells/sample) were seeded in a tissue-culture treated polymer chamber (Ibidi USA) in either BMDC media [RPMI 1640 (Corning) with 10% FBS, 1 ​mM sodium pyruvate (HyClone), 2 ​mM l-glutamine (Corning), 100 U/mL Pen/Strep (Gibco), 50 ​mM 2-Mercaptoethanol (Gibco), and 0.1 ​mM nonessential amino acids (Lonza)] or macrophage media (previously defined), and allowed to settle for 20–24 ​h at 37 ​°C.

Lysotracker Green DND-26 (Invitrogen) (50 ​nM) was added to the chambers and incubated with the cells for at least 1 ​h at 37 ​°C to locate the acidic compartments. Excess Lysotracker was removed by washing cells with media three times. It was previously shown by our group that significant levels of E2 uptake is observed in dendritic cells and macrophages after 1 ​h of incubation [44]. Thus, 400 ​nM NLuc on NLuc-E2 nanoparticles were incubated with BMDCs for an hour to ensure the time was sufficiently long for NLuc-E2 uptake. Cells were washed with media at least five times to reduce the background noise. 200 ​μL of a furimazine solution (1:100) diluted in media was added to chambers, and the bioluminescence from NLuc-E2 was tracked for up to 35 ​min. Immediately afterwards, the fluorescent images from Lysotracker were captured using the same microscope. For the duration of imaging, cells were maintained at 37 ​°C supplemented with 5% CO_2_. Samples were excited with a 491 ​nm laser (Olympus) for Lysotracker, and the exposure times for bioluminescence and fluorescence imaging were 10 ​s and 10 ​ms respectively. All images were acquired with a 20X air objective (Zeiss UPlanSAPO ×20/0.75) with further x2 magnification. The bioluminescent and fluorescent images were shown separately or merged after processing.

Alexa Fluor 568 C_5_-Maleimide (AF568) was conjugated to the interior cysteines of NLuc-E2 following the same conjugation methods used for conjugating Alexa Fluor 750 C_5_-maleimide (Details in Supplemental Methods). The same incubation conditions described above for Lysotracker staining were followed, and 100 ​nM of NLuc on AF568-NLuc-E2 were incubated with RAW 264.7 macrophage cells for an hour followed by removal of residual AF568-NLuc-E2 by gently washing with media. The fluorescence from AF568 and Lysotracker was imaged and the images were merged together. A SuperK Evo White Light Laser (NKT Photonics) with excitation filter 560/15 (Chroma) was used for imaging AF568-NLuc-E2.

### 2.8 *In vivo* tracking of imaging molecules and constructs

All animal studies were carried out under protocols approved by the Institute of Animal Care and Use Committee (IACUC) at the University of California, Irvine. Female B6(Cg)-Tyr^c−2J^/J (6–8 weeks) mice (albino) were purchased from the Jackson Laboratory. Mice were injected subcutaneously at base of the tail with 4 ​μM NLuc free in solution or on nanoparticles (NLuc-E2), or controls [including PBS negative control, an equal molar amount of E2 nanoparticles in E2-AF750 (AF-E2), or an equal concentration of free AF750 (AF)] diluted in PBS. The AF concentration used in the injection was ∼11 ​μM. Two 50 ​μL injections were administered at either side of the base of the tail (defined as 0 ​h), and the mice were shaved around the injection sites before imaging. Mice were sedated under isoflurane during imaging, and the fluorescence or bioluminescence was tracked with IVIS Lumina system. Mice were imaged *in vivo* at 0, 3, 24, 48, or 72 ​h in dorsal and ventral positions. Fluorescent images were acquired with the appropriate filters for AlexaFluor 750, with an excitation laser of 745 ​nm and emission filter passband of 810–875 ​nm 100 ​μL of a 1:20 dilution of furimazine in PBS was injected intraperitoneally for the PBS, NLuc-E2, and NLuc treatment mice. Bioluminescent images were acquired when the luminescence reached steady-state following furimazine injection (∼10 ​min after). Bioluminescent images were acquired with block excitation and open emission settings. The exposure times for fluorescence and bioluminescence imaging were set as auto to avoid oversaturation. Mice were sacrificed at day 1 and 3, and *ex vivo* imaging of organs was performed following collection of blood through heart puncture. The heart, lungs, liver, spleen, intestines, kidneys, inguinal LNs, injection sites, mesenteric LNs, and axillary LNs were harvested for each mouse. The organs were rinsed with PBS and soaked in PBS containing furimazine (1:100) for 5 ​min. All organs were patted dry before imaging. After imaging, each organ was weighed for data normalization. Extracted blood was allowed to clot at room temperature for at least 1 ​h and was centrifuged at 2000×*g* for 10 ​min. 25 ​μL of serum from each mouse was placed in a 96-well clear bottom black plate, and 25 ​μL of 1:100 furimazine in PBS was added to the serum obtained from PBS, NLuc-E2 or NLuc treatment mice. The fluorescence or bioluminescence of the serum was measured with IVIS using the same settings described above. The entire mouse in dorsal or ventral view or the entire organ was specified as the region of interest for the *in vivo* and *ex vivo* imaging quantification. The total flux or total radiant efficiency for *in vivo* and *ex vivo* imaging was quantified using Living Image software.

### 2.9 Statistical analysis

The statistical analyses were performed with GraphPad Prism. We utilized one-way or two-way ANOVA with Tukey's multiple comparison test over the experimental groups, as described in the figure captions. The data were shown as average ​± ​standard error of mean (SEM), and n ​≥ ​3 for all experiments, unless stated otherwise. In all cases, p ​< ​0.05 is considered statistically significant.

## 3 Results and discussion

### 3.1 NLuc and Akaluc conjugated to E2 using SpyCatcher-SpyTag resulted in intact and monodisperse nanoparticles

**Fig. 1:**
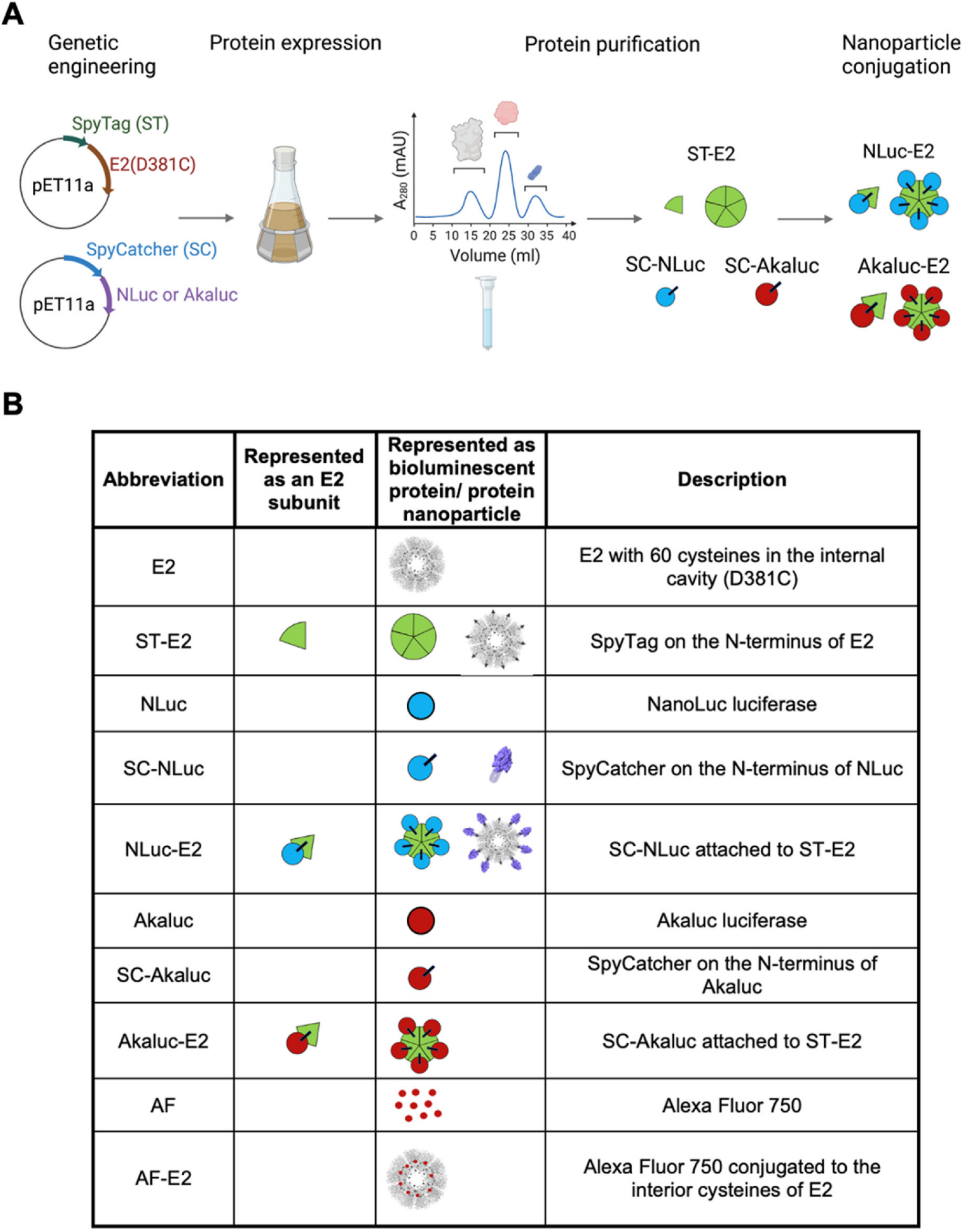
Summary of syntheses and abbreviations of protein and nanoparticle components. (A) Schematic of nanoparticle synthesis, from genetic engineering to nanoparticle conjugation with bioluminescent molecules. ST-E2, SC-NLuc and SC-Akaluc were recombinantly expressed in *E. coli* and purified. ST-E2 and SC-NLuc or SC-Akaluc were mixed to generate NLuc-E2 and Akaluc-E2. (B) List of the components used in this study with their respective descriptions. It should be noted that E2 consists of 60 subunits, and the green wedge represents one E2 subunit whereas the circular assembly represents an E2 nanoparticle.

NLuc and Akaluc were chosen as the enzymes for immobilization to SpyTag-E2 (abbreviated ST-E2). ST-E2, SC-NLuc, SC-Akaluc, NLuc, and Akaluc were made recombinantly, and the NLuc and Akaluc were attached to E2 through the SC-ST approach, yielding SpyCatcher-NLuc (SC-NLuc) and SpyCatcher-Akaluc (SC-Akaluc), respectively (Fig. 1A). All the components and samples used in this study are summarized (Fig. 1B).

**Fig. 2:**
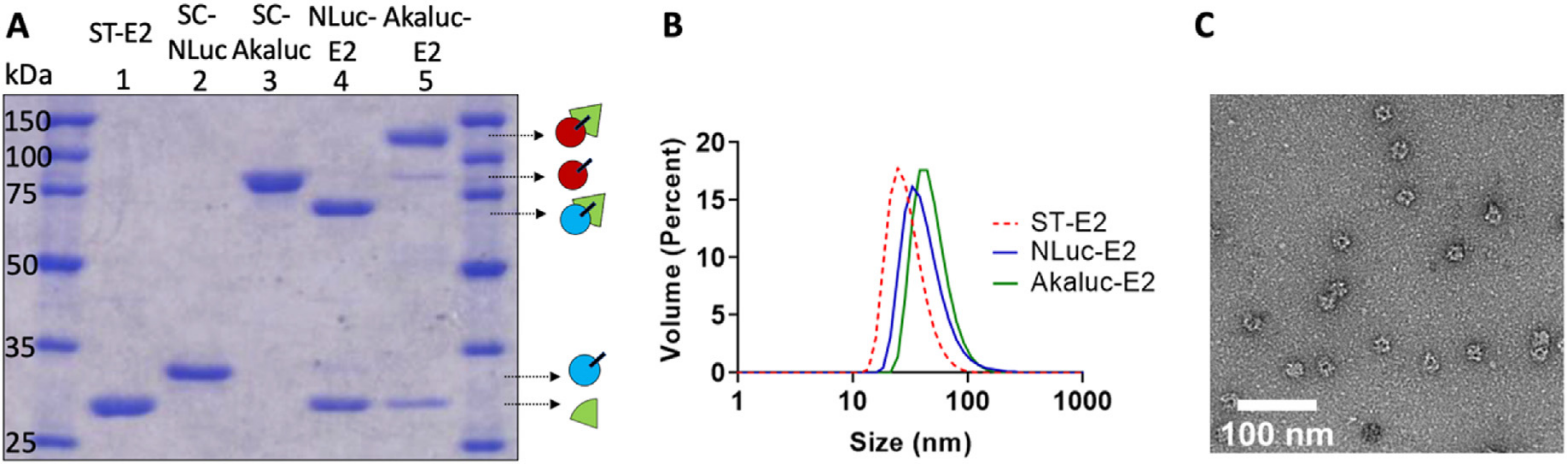
Characterization of NLuc-E2 and Akaluc-E2. (A) SDS-PAGE of nanoparticle components confirms attachment of NLuc and Akaluc to E2. Lanes: 1. ST-E2; 2. SC-NLuc; 3. SC-Akaluc; 4. NLuc-E2; and 5. Akaluc-E2. (B) Representative hydrodynamic diameters of nanoparticles (ST-E2, NLuc-E2, and Akaluc-E2). (C) TEM image of NLuc-E2 nanoparticles. Scale bar ​= ​100 ​nm.

SDS-PAGE showed that the monomers of NLuc-E2 and Akaluc-E2 were at the expected sizes of 65 ​kDa and 108 ​kDa, respectively (Fig. 2A). We obtained conjugation ratios of 23.1 ​± ​1.4 NLuc molecules per E2 nanoparticle and 20.5 ​± ​2.1 Akaluc molecules per E2 nanoparticle. Given that the attachment of SC-NLuc or SC-Akaluc to ST-E2 appears to be saturated at mixture ratios of 0.7 NLuc:1 E2 monomer and 0.6 Akaluc:1 E2 monomer, respectively (Figure SI-1), and the ST-SC conjugation is known to be >80% efficient [19], we speculate that attachment of NLuc and Akaluc may be sterically hindered. Although a previous study reported complete conjugation using the ST-SC system with the E2 nanoparticle [45], the attached entity was an elastin-like polypeptide (ELP), which is considered to be an intrinsically disordered protein polymer [46] and does not have the stricter geometric and steric constraints as a folded enzyme such as NLuc.

Dynamic light scattering showed that nanoparticle sizes were 43.6 ​± ​1.5 ​nm for NLuc-E2 and 47.2 ​± ​1.7 ​nm for Akaluc-E2. These values are consistent with an increase in size from 30.0 ​± ​1.3 ​nm for ST-E2 (no bioluminescent protein) (Fig. 2B), which is slightly larger than the E2 without the SpyTag [10]. NLuc-E2 was imaged with transmission electron microscopy (TEM), which further demonstrated that the nanoparticles were intact (Fig. 2C). These results support the decoration of the bioluminescent proteins around the surface of E2 and demonstrate that the 60-mer protein assemblies remained intact after conjugation.

### 3.2 NLuc attached to nanoparticle (NLuc-E2) showed over two orders of magnitude higher luminescence at pH 5.0 than unconjugated NLuc

**Fig. 3:**
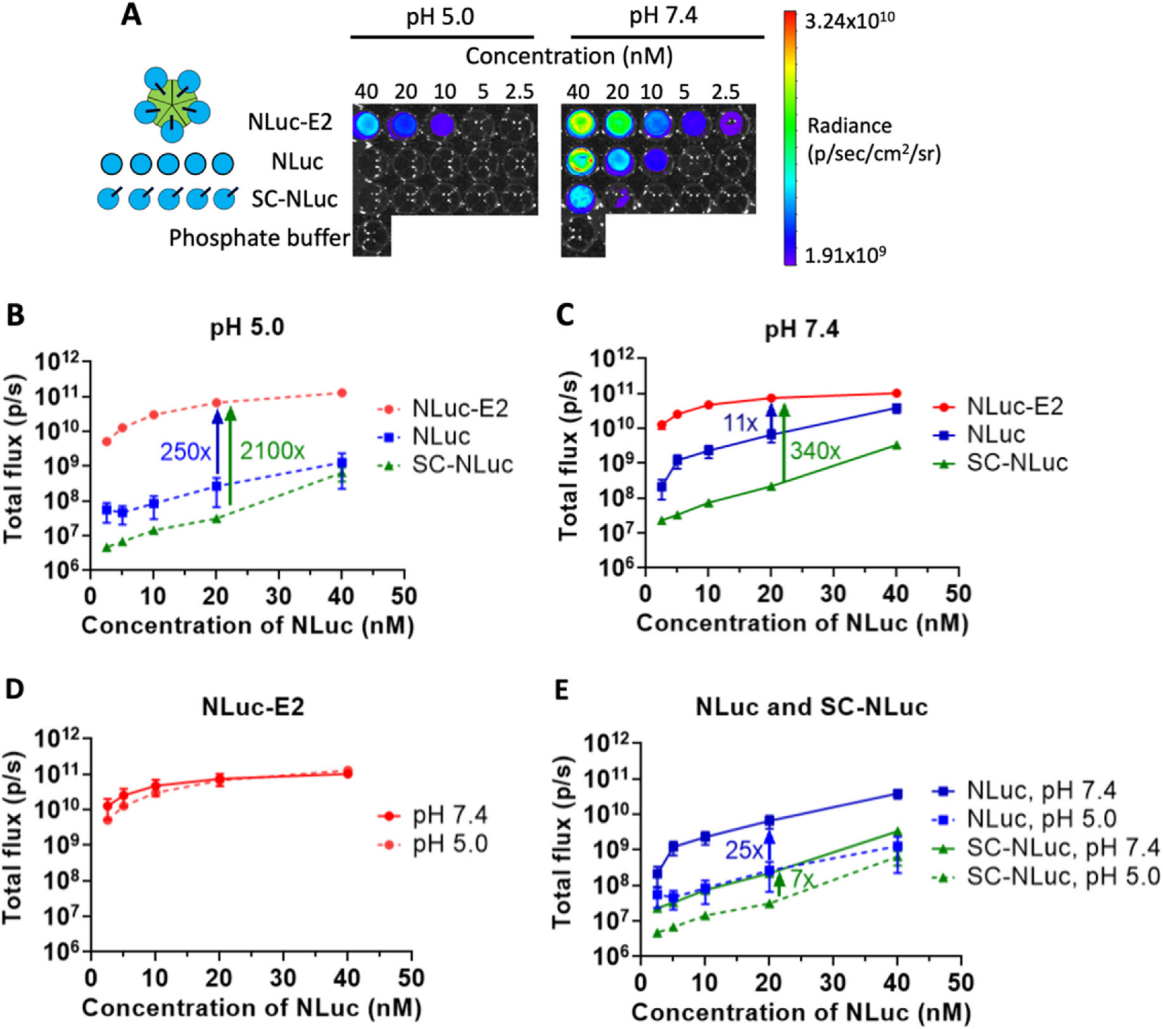
Luminescence of NLuc constructs at pH 5.0 (dashed lines) and pH 7.4 (solid lines). (A) NLuc-E2, NLuc, SC-NLuc, and phosphate buffer at pH 5.0 and pH 7.4 were imaged at different concentrations. (B) At pH 5.0, bioluminescent light emission by NLuc-E2 (red dots), NLuc (blue squares), and SC-NLuc (green triangles) at different concentrations, and the fold-difference between NLuc-E2 and either NLuc or SC-NLuc at pH 5.0, is shown. (C) At pH 7.4, bioluminescent light emission by NLuc-E2, NLuc, and SC-NLuc at different concentrations, and the fold-difference between NLuc-E2 and either NLuc or SC-NLuc at pH 7.4, is shown. (D) Luminescence of NLuc-E2 vs. concentration, at pH 5.0 and 7.4. Free NLuc-E2 showed comparable luminescence at the two pH levels. (E) Luminescence of free NLuc and SC-NLuc vs. concentration, at pH 5.0 and pH 7.4, and the fold-difference of luminescence at pH 5.0 and pH 7.4 for NLuc (blue) or SC-NLuc (green). The total flux for each condition was measured and plotted as average ​± ​SEM of n ​= ​3 independent experiments.

**Fig. 4:**
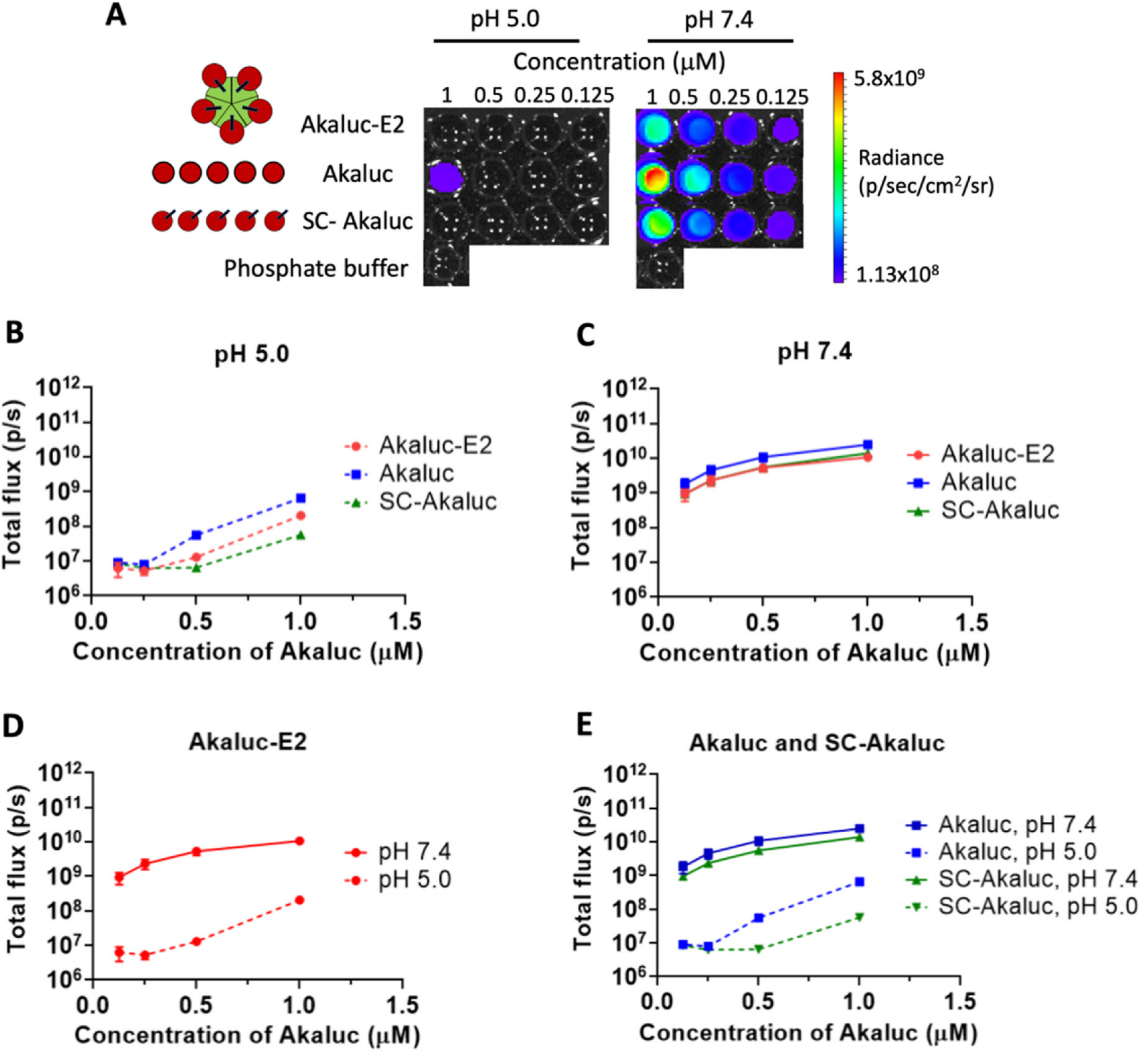
Luminescence of Akaluc constructs at pH 5.0 (dashed lines) and pH 7.4 (solid lines). (A) Akaluc-E2, Akaluc, SC-Akaluc, phosphate buffer at pH 5.0 and 7.4 were imaged at different concentrations. (B) Bioluminescence of Akaluc-E2 (red dots), Akaluc (blue squares), and SC-Akaluc (green triangles) at different concentrations at pH 5.0 was quantified. (C) Bioluminescence of Akaluc-E2, Akaluc, and SC-Akaluc at different concentrations at pH 7.4 was measured. (D) Bioluminescent light emission of Akaluc-E2 at pH 5.0 and pH 7.4. (E) Luminescence of Akaluc and SC-Akaluc at pH 5.0 and pH 7.4. The total flux for each condition was measured and plotted as average ​± ​SEM of n ​= ​3 independent experiments.

We were interested in the effects of nanoparticle uptake by cells on bioluminescence, and since endosomal/lysosomal compartments exhibit acidic pH conditions, we examined whether pH would affect the bioluminescence of constructs. Therefore, we compared the intensities of nanoparticle-bound bioluminescent proteins (NLuc-E2, Akaluc-E2) and those which were not attached to nanoparticles (SC-NLuc, SC-Akaluc, NLuc, Akaluc) at pH 7.4 and pH 5.0 (Fig. 3, Fig. 4).

*Bioluminescence at* pH *5.0.* Interestingly, NLuc-E2 demonstrated 250-fold and 2100-fold higher luminescence than NLuc and SC-NLuc, respectively, under acidic conditions at 20 ​nM of NLuc. The increased signal for nanoparticle-bound NLuc was observed across all concentrations that were examined (Fig. 3A and B). This suggests that luminescence can still be detected when NLuc-E2 enters the acidic endo-lysosomal compartments of the cells after uptake. There is no significant difference (*p* ​> ​0.05) between NLuc and SC-NLuc at pH 5.0, both of which involve NLuc that are not bound to the nanoparticle. Furthermore, NLuc-E2 showed comparable luminescence between pH 7.4 and pH 5.0 (Fig. 3D), whereas NLuc and SC-NLuc (both without nanoparticles) showed a decrease of 25-fold and 7-fold in luminescence at 20 ​nM in pH 5.0 phosphate buffer (Fig. 3E). These pH-dependent trends are also observed in different buffers (Figure SI-2A).

*Bioluminescence at* pH *7.4.* At pH 7.4, the luminescence of NLuc at 20 ​nM was ∼6.5 ​× ​10^9^ p/s, but attaching a SpyCatcher to this molecule (SC-NLuc) decreased the value by approximately 30-fold (2.2 ​× ​10^8^ p/s) when compared to NLuc at 20 ​nM (Fig. 3C). However, by immobilizing SC-NLuc onto the E2 nanoparticle (NLuc-E2), the luminescence of NLuc-E2 showed an 11-fold and 340-fold increase in luminescence compared to NLuc and SC-NLuc, respectively (Fig. 3C). It should be noted that although the maximum emission wavelength of NLuc on the nanoparticles (NLuc-E2) did not shift compared to either NLuc or SC-NLuc, the overall spectra is broader and red-shifted (Figure SI-3). This result of increased luminescence after nanoparticle conjugation at pH 7.4 is also reported in other publications, although exact fold increases vary; for example, a 2.7-fold increase of luminescence was reported after ST-NLuc was conjugated to a SC-hepatitis B virus-like particle [24]. NLuc has also been attached to E2 using an alternative attachment strategy (sortase A) [47], although the authors attributed the observed five-fold increase in luminescence to the corresponding five-fold higher amount of NLuc attached to the E2 nanoparticles. An approximate 10-fold increase was observed after displaying multiple NLuc on a protein nanoparticle consisting of elastin-like polypeptides [48]. This could be explained by the enhanced affinity between the NLuc and substrate from the multivalent interactions, as papers have demonstrated up to 4 orders of magnitude increase of target-specific avidity after multiple targeting ligands (FK506) are conjugated on the magnetic nanoparticle [49].

We also observed that the surface density of the immobilized NLuc can significantly affect the intensity of luminescence. The luminescence of 0.2:1 and 0.7:1 SC-NLuc to ST-E2 molar conjugation ratios were tested to represent a lower and higher density of NLuc per E2 (while keeping total NLuc concentration constant). We obtained a conjugation ratio of 12 ​± ​1.2 NLuc molecules per E2 nanoparticle for the 0.2:1 SC-NLuc to ST-E2 ratio. The higher density of NLuc attached to a nanoparticle surface (0.7:1 SC-NLuc to ST-E2 ratio) showed a significant increase in luminescence by ∼1.8-fold compared to NLuc-E2 with lower NLuc density (0.2:1 SC-NLuc to ST-E2 ratio) (*p* ​< ​0.01; Figure SI-4). However, the lower density of NLuc attached onto ST-E2 still showed significantly higher luminescence than free SC-NLuc, confirming that immobilizing NLuc on the nanoparticle and the proximity of the multiple NLuc molecules on the nanoparticle surface can increase luminescence even if the density of the immobilized enzyme is relatively low.

There are many possible explanations for the enhancement of bioluminescent performance when NLuc is conjugated to a nanoparticle at these physiological pH conditions. Studies have suggested that the enzyme orientation is an important factor in potentially increasing the likelihood of enzyme-substrate interactions [12,13,50]. NLuc and furimazine interactions are computationally suggested to be driven by polar and hydrophobic interactions at amino acid residues Q32, S37, L18, L22, F31, P40, V58, and F110 on NLuc [51]. The SC attached on the N-terminus is on the opposite side of NLuc relative to the active site (Figure SI-5), which should keep all the active sites facing outward, enabling accessibility of the active sites while potentially blocking non-catalytic interactions of the substrate with NLuc [38]. Another possible explanation is that the pH of the local microenvironment surrounding NLuc-E2 could be different than the bulk solution for free NLuc, which could change stability or the efficacy of the catalytic function. It has been hypothesized that the scaffolds onto which enzymes are conjugated can shift the local pH microenvironment around the enzymes [52,53]. Zhang *et al.* [54] suggested that negatively-charged DNA in alkaline pH can decrease the local pH because the protons in the bulk solution are attracted to the negatively-charged DNA surface, leading to a more optimal pH for an immobilized GOx-HRP cascade. Others have suggested that gold nanoparticles can act as a buffer system, where they attract protons at acidic conditions and repulse protons under alkaline conditions [55]. For our studies here, ST-E2 was calculated to have an isoelectric point (pI) near 8.5 [37], and NLuc and SC-NLuc have pIs of 4.9 and 4.8, respectively [37]. Thus, at pH 5.0, the local pH around the positively charged ST-E2 could possibly be less acidic than in the bulk solution, creating a more suitable local pH for SC-NLuc to carry out its enzymatic performance. Another possible explanation is that conjugation between ST-E2 and SC-NLuc can help reduce any destabilizing conformation changes of NLuc at pH 5, making the immobilized SC-NLuc on ST-E2 more stable and tolerant to the acidic pH conditions [56,57].

### 3.3 Akaluc attached to E2 (Akaluc-E2) retained its bioluminescence after conjugation to nanoparticle

For Akaluc, we found that all the constructs demonstrated over two orders of magnitude higher luminescence at pH 7.4 compared to pH 5.0 (Fig. 4A, D, 4E, SI-2B). Furthermore, free Akaluc showed slightly higher average luminescence than SC-Akaluc and Akaluc-E2 at both pH conditions (Fig. 4B and C). Attaching SpyCatcher to the N-terminus of Akaluc caused a slight decrease in luminescence, giving an approximately 2-fold decrease in luminescence at 0.5 ​μM ​at pH 7.4 (Fig. 4C). However, immobilizing SC-Akaluc onto ST-E2 showed comparable luminescence to free SC-Akaluc alone. Thus, the immobilization of SC-Akaluc on ST-E2 enabled Akaluc to retain its luminescent performance at neutral pH. It should be noted that the relevant concentrations for Akaluc samples are at the order of μM, and NLuc samples are at the nM level. However, the two bioluminescent proteins are showing similar orders of radiance, suggesting that NLuc is brighter than Akaluc under the same protein concentration. This is consistent with studies that have shown that photon emission from NLuc with furimazine is approximately 150-fold higher than firefly luciferase with D-luciferin, and firefly luciferase with D-luciferin generates higher luminescence than Akaluc with Akalumine-HCl [14,58].

These results imply that Akaluc-E2 can be utilized in *in* *vivo* imaging applications that require red-shifting luminescence at neutral pH. Together with the NLuc data, this also suggests that the enhanced bioluminescent performance after attachment to a nanoparticle can be enzyme dependent [59]. Since we are interested in examining the luminescence of constructs after cell uptake, the *in vitro* and *in vivo* studies in the following sections were performed with NLuc and NLuc-E2.

### 3.4 Luminescence of NLuc-E2 nanoparticle persisted longer than free NLuc after macrophage uptake

**Fig. 5:**
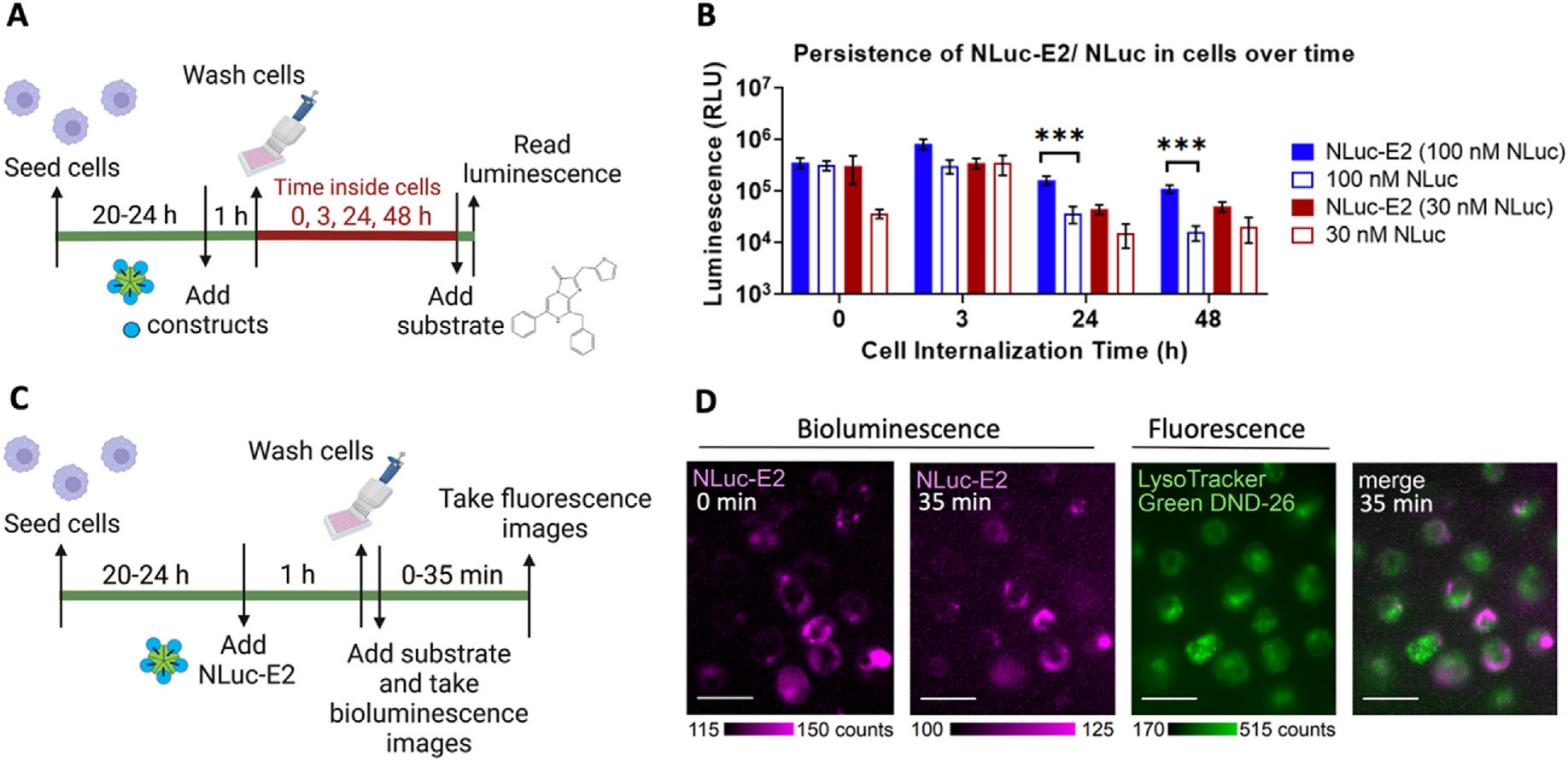
Persistence of NLuc-E2 or NLuc in antigen-presenting cells over time. (A) Schematic of the experimental methods used to measure the luminescence in RAW 264.7 macrophages over time. (B) Measurement of luminescence in cells over time. Cells were incubated with the same molar concentrations of NLuc (in NLuc-E2 and NLuc) at 30 ​nM or 100 ​nM for 1 ​h uptake, washed thoroughly, and further incubated for cell processing. Values are expressed as average ± SEM; n ​≥ ​5. Significance was determined between bound and unbound groups at the same timepoint with one-way ANOVA followed by Tukey's multiple comparisons test (∗∗∗*p* ​< ​0.001). (C) Schematic of the workflow of live cell imaging. (D) Nanoparticles with dendritic cells were imaged with a bioluminescent microscope and tracked over time. Bioluminescence from NLuc-E2 (magenta) was followed from 0 to 35 ​min after uptake, fluorescence from Lysotracker (green) was imaged at 35 ​min, and the images were merged. Counts represent intensity counts, and scale bars ​= ​25 ​μm.

To utilize NLuc-bound nanoparticles for *in vitro* and *in vivo* imaging, their luminescence properties when internalized by cells is important. Thus, we evaluated whether NLuc-E2 could still be detectable over time after cell uptake. A macrophage-like cell line (RAW 264.7) was chosen for uptake studies because their mechanisms of uptake include pinocytosis and phagocytosis [60]. Details of these studies are described in Fig. 5A and Methods. After incubation of cells with the same molar concentrations (30 or 100 ​nM) of NLuc in the form of NLuc-E2 or free NLuc and washing off the excess non-internalized NLuc-E2 or NLuc, the length of time of NLuc-E2 or NLuc within the cell (“Time inside cells”) was varied and the resulting luminescence was measured (Fig. 5A).

We observed that the luminescence from NLuc-E2 persisted longer than free NLuc in cells after uptake. At the initial timepoint (t ​= ​0 ​h), 100 ​nM NLuc-E2 and NLuc had similar levels of luminescence; however, the NLuc luminescence decreased over time, and by 24 ​h became significantly lower than NLuc-E2 (Fig. 5B). At 24 and 48 ​h, in cells incubated with a total NLuc concentration of 100 ​nM, NLuc-E2 had a significantly higher luminescence than those incubated with free NLuc. Although there was no statistical difference between the groups that received 30 ​nM NLuc (in NLuc-E2 and free NLuc), the mean luminescence with NLuc-E2 was greater than with free NLuc (Fig. 5B). We have previously demonstrated that E2 nanoparticles can be taken up by dendritic cells, macrophages, and cancer cells, likely through the endocytic pathway [44,61]. Since this pathway involves acidic endosomal (∼pH 6.5) and lysosomal (pH 4.5) compartments [34], based on data shown in Fig. 3B, luminescence from NLuc alone should be lower than from NLuc-E2 once it enters the acidic compartments. The observation of longer luminescent persistence in Fig. 5B is consistent with what we expected and would be a favorable result towards the signal and stability for *in vivo* tracking of NLuc-E2 over time. We note that the decrease in luminescence for both NLuc-E2 and NLuc between 0 and 24 ​h is likely not due to thermal instability of samples at 37 ​°C, as NLuc-E2 and NLuc alone in media at 37 ​°C did not show significant difference between 0 and 24 ​h for all the concentration tested (Figures SI-6A and SI-6B).

We then examined whether NLuc-E2 could be imaged in live cells after uptake. We used a camera-based bioluminescence/fluorescence microscope to capture the bioluminescence from NLuc-E2 after 1 ​h of incubation (Fig. 5C and D), and fluorescent Lysotracker Green DND-26 was utilized to label the acidic compartments of the cells. Dendritic cells and macrophages were chosen for live cell imaging as they are known to internalize E2 efficiently [44]. We showed that cells with NLuc-E2 could be imaged with a bioluminescence microscope, together with the fluorescence imaging component for Lysotracker (Fig. 5D), although these regions do not appear to be co-localized. AF568 was also conjugated to the interior of NLuc-E2 (AF568-NLuc-E2), and fluorescence imaging suggest that cells were taking up E2 (Figure SI-7), with regions of punctate AF568 colocalizing with the endosome/lysosome. Although further investigations are needed to elucidate the modes of uptake and processing, these data show that the bioluminescence emitted from the NLuc-E2 particle can be used to image live cells using microscopy.

### 3.5 Non-invasive *in vivo* imaging of biodistribution showed higher signal-to-noise ratios over time using NLuc-E2 nanoparticles than unbound NLuc and fluorescently-labeled E2 nanoparticles

To assess the feasibility of using NLuc-E2 for *in* *vivo* imaging and biodistribution studies, mice were administered with NLuc-E2 and imaged over 72 ​h. AlexaFluor 750 (AF) conjugated to the interior cysteines of E2 (D381C form of E2, without SpyTag; Figure SI-8) served as a point of comparison (AF-E2), together with additional controls of the molecules not attached to nanoparticles (free NLuc and AF) and PBS.

**Fig. 6:**
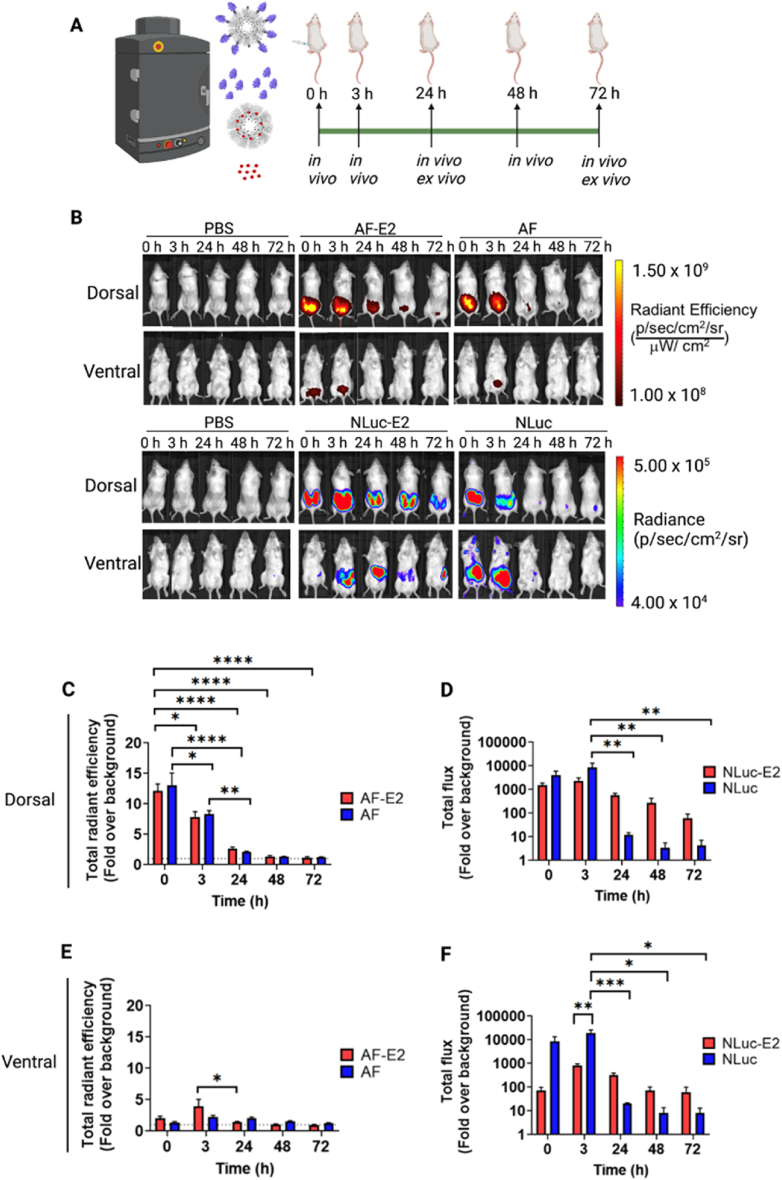
*In vivo* tracking of the biodistribution of fluorescent and bioluminescent constructs. Solutions of PBS, AF-E2, AF, NLuc-E2 and NLuc were subcutaneously injected into mice, and the fluorescence (PBS, AF-E2, AF) or bioluminescence (PBS, NLuc-E2, NLuc) was tracked over time. (A) Schematic of the experimental timeline. (B) Representative fluorescent and bioluminescent images of live imaging with dorsal and ventral views are shown. (C) Quantification of fluorescence from dorsal view. (D) Quantification of bioluminescence from dorsal view. (E) Quantification of fluorescence from ventral view. (F) Quantification of bioluminescence from ventral view. Data are represented as fold over the fluorescence or bioluminescence obtained from the PBS-injected mice at time 0 (background). The dotted lines indicate normalized PBS value at 1. Values are expressed as average ± SEM for n ​≥ ​3 for all groups, except for AF at 48 and 72 ​h (which were n ​= ​2). Two-way ANOVA with Tukey's multiple comparison test were performed (∗*p* ​< ​0.05, ∗∗*p* ​< ​0.01, ∗∗∗*p* ​< ​0.001, ∗∗∗∗*p* ​< ​0.0001).

**Fig. 7:**
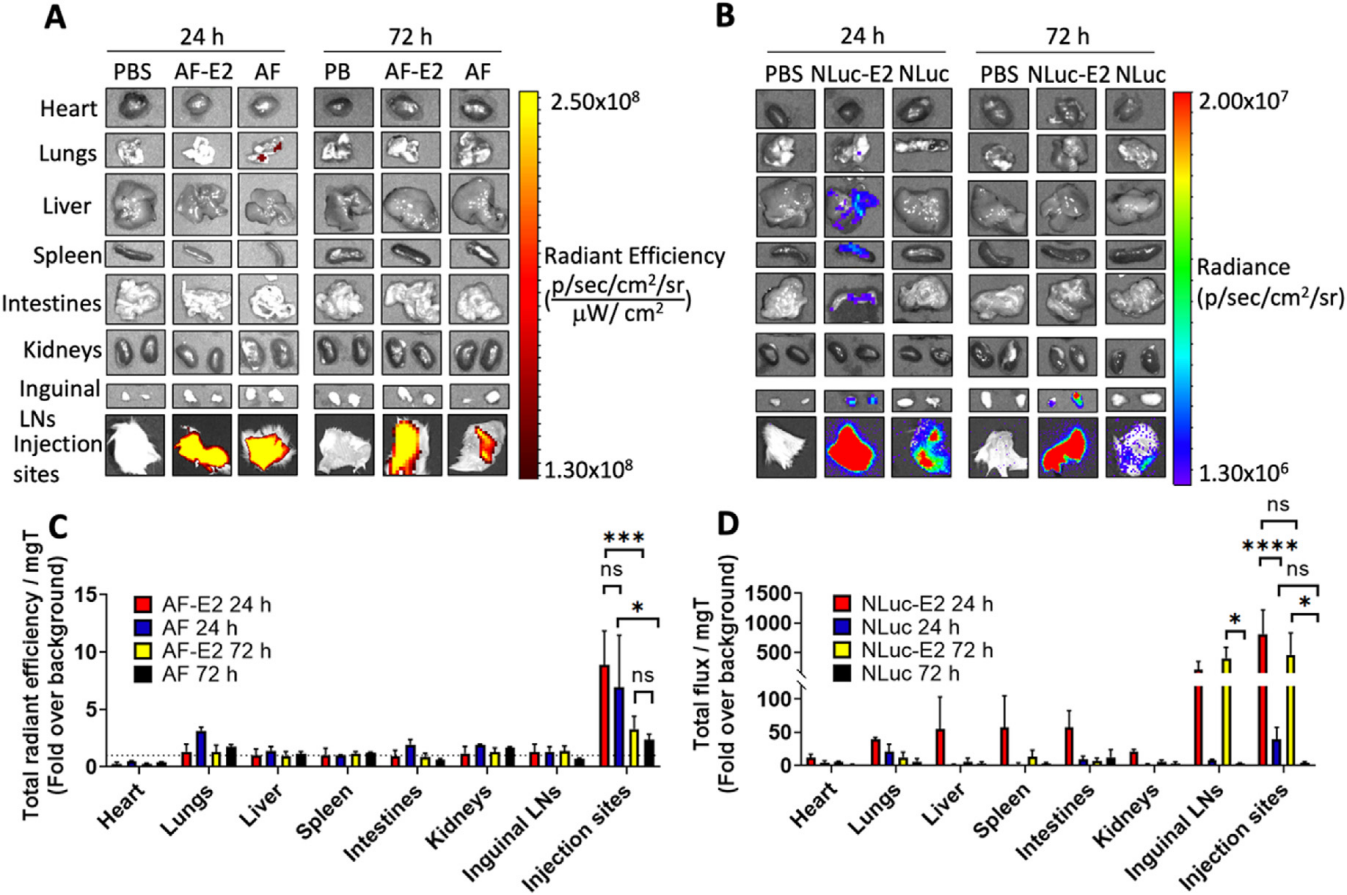
Elucidating the locations of the *in vivo* light emissions using *ex vivo* imaging. (A) Representative fluorescent *ex vivo* images of the harvested organs (heart, lungs, liver, spleen, intestines, kidneys, inguinal LNs, and injection sites) on day 1 and day 3 from PBS, AF-E2, and AF mice. (B) Representative bioluminescent *ex vivo* images of the harvested organs on day 1 and day 3 from PBS, NLuc-E2, and NLuc mice. (C) Fluorescence from the *ex vivo* images. The dotted lines indicate normalized PBS value at 1. (D) Bioluminescence from the *ex vivo* images. Regions of interest were drawn for each organ and the total fluorescence or bioluminescence was normalized to milligrams of tissue. Finally, the data were reported as fold over fluorescence or bioluminescence obtained from PBS-injected mice. Values are expressed as average ± SEM for n ​= ​3 individuals. Two-way ANOVA with Tukey's multiple comparison test were performed (∗*p* ​< ​0.05, ∗∗*p* ​< ​0.01, ∗∗∗*p* ​< ​0.001, ∗∗∗∗*p* ​< ​0.0001).

*NLuc-E2 persisted longer than AF-E2 and NLuc when imaging in vivo.* All of the groups were imaged in real-time at 0, 3, 24, 48, and 72 ​h under dorsal and ventral views (Fig. 6), and *ex vivo* imaging of organs and tissues was performed at 24 and 72 ​h (Fig. 7). The experimental flow is shown in Fig. 6A and Methods. The dorsal images from AF-E2 and AF show bright fluorescence around the injection sites at time 0 that generally decreased over time (Fig. 6B and C). NLuc-E2 persisted much longer than both NLuc (starting from 24 ​h, in the dorsal view) (Fig. 6D) and AF-E2 (Fig. 6C). AF-E2 ventral views yielded even lower fluorescent signal (Fig. 6E). Overall, the biodistribution of AF-E2 is unclear from the *in vivo* images. In contrast, light emission from NLuc-E2 was observed across all the time points in the *in vivo* images from both the dorsal and ventral views (Fig. 6B). Similar to AF-E2, NLuc-E2 signal decreased over time. Interestingly, NLuc-E2 was observed in the upper abdominal section in the ventral view starting from 3 ​h and lasted for 72 ​h (Fig. 6B and F). The bioluminescence was postulated to emanate from the inguinal lymph nodes (LNs), which was later supported with *ex vivo* imaging (Fig. 7).

NLuc-E2 could be tracked longer than AF-E2 over time, likely because NLuc-E2 is brighter than AF-E2, and has better signal-to-noise ratios than AF-E2 *in vivo*. From the dorsal view, signal from NLuc-E2 was ∼1550-fold above background at 0 ​h, 570-fold above background at 24 ​h, and significantly, at 72 ​h, NLuc-E2 was still ∼60-fold above background (Fig. 6D). In contrast, AF-E2 showed only 12-fold over background at 0 ​h, 3-fold higher above background at 24 ​h, and no difference from background at 72 ​h (Fig. 6C). The ventral view of NLuc-E2 also demonstrated ∼320 and ∼60-fold increase above background at 24 ​h and 72 ​h, respectively, while AF-E2 was close to background starting at 24 ​h (Fig. 6E and F). In addition to NLuc-E2 exhibiting better signal-to-noise ratios over AF-E2, NLuc-E2 also demonstrated higher luminescence than NLuc over time in both ventral and dorsal views. Since we speculate that the locations of the most intense luminescence from the images starting from 24 ​h are from the injection sites and inguinal LNs (and later supported by *ex vivo* imaging), this suggests that NLuc-E2 persists longer at the injection sites and inguinal LNs than NLuc.

In summary, our results show that NLuc-E2 emission signal is up to ∼1000-fold above background (Fig. 6D and F), while AF-E2 is up to ∼10-fold above background within the imaging time points (Fig. 6C and E). This can be explained by red-shifted fluorescent dye generally yielding lower light emission [62]. Furthermore, the 150-Watt quartz halogen lamp in the IVIS imager [63] as the fluorescence excitation light source might not be powerful enough for AF-E2 for long-term *in vivo* imaging. Therefore, NLuc-E2 can be a better imaging platform than AF-E2 for non-invasive biodistribution studies *in vivo*.

*Light emission signal was observed in draining lymph nodes (LNs) for bioluminescent E2 but not for fluorescent E2, demonstrating significantly higher sensitivity of imaging with bioluminescent nanoparticles.* Tissues were harvested and imaged *ex vivo* to examine the biodistribution with fluorescence (Fig. 7A) and bioluminescence (Fig. 7B) and results were correlated with the *in vivo* images (Fig. 6B). Fluorescence was observed in the injection sites for both AF-E2 and AF up to 72 ​h (Fig. 7C), and a low level of fluorescence was detected in the lungs for mice with AF at 24 ​h. However, the rest of the organs, including inguinal LNs for AF-E2 and AF, showed fluorescence close to background. In contrast, we observed luminescence from NLuc-E2 in the liver, spleen, inguinal LNs, and injection sites (Fig. 7B and D). Interestingly, the inguinal LNs and injection sites showed the highest signals of all the organs, demonstrating up to 1000-fold above background, and remained strong at 72 ​h. These signals are consistent with high amounts of E2 which naturally distribute into these tissues [44]. These tissues could correlate to the bioluminescence seen in the ventral *in vivo* images for the NLuc-E2 mice (Fig. 6D). Similar to the observation from the *in vivo* imaging, the luminescence observed in the mice which were administered free NLuc (no nanoparticle) at the injection sites was significantly lower than NLuc-E2 at both 24 and 72 ​h.

In addition to observing very strong signal in the inguinal LNs, bioluminescence was also detected in the axillary and mesenteric LNs for NLuc-E2 mice (Figures SI-9A and SI-9B), signals of which are ∼60 and 900-fold higher than background, respectively (Figure SI-9D). However, neither of these LNs from the mice that were administered the fluorescence AF-E2 validated the presence of the particles, with fluorescence close to background (Figure SI–9C). Furthermore, bioluminescence was detected in the sera of the mice that were administered NLuc-E2 or NLuc (Figures SI-10B and SI-10D). Sera from mice that were given NLuc-E2 had higher bioluminescence than NLuc at both 24 and 76 ​h, suggesting that NLuc-E2 has a longer elimination half-life than NLuc. This could be due to the size difference of NLuc-E2 and NLuc, thereby impacting how APCs interact with them [30], their biodistribution, and the kinetics of biodistribution. Although NLuc shows an elevated signal when bound to E2 (Fig. 3C), this ∼10-fold difference alone is not enough to account for the higher differences seen in the sera (Figure SI-10). In contrast, fluorescence detected in the serum of the AF-E2 and AF mice was close to background, possibly due to detection limitations and poorer signal-to-noise ratios relative to bioluminescence (Figures SI-10A and SI-10C).

We previously confirmed and quantified the distribution of the E2 particles to the draining LNs using flow cytometry, and ∼50% of the dendritic cells (DCs) and macrophages in the draining LNs were associated with (fluorescently-labeled) E2 [44]. Inguinal and axillary LNs are the draining LNs for base of the tail injections [64], and in these current imaging studies, we did observe the NLuc-E2 signals for both types of LN but not for fluorescently-labeled E2. This could be attributed to the amount of E2 accumulated in the LNs being below the detection limit using IVIS, and higher amounts of AF-E2 might be needed for tracking *ex vivo*. Although NLuc emits light at 460 ​nm and the emitted light is difficult to be detected in the deeper organs [14,15], the luminescence of NLuc was sufficiently bright to allow a considerable amount of bioluminescence from NLuc-E2 to penetrate through the animals, yielding more sensitivity than AF-E2.

It may be possible to obtain further enhancement of *in vivo* tissue penetration using alternative systems. For example, the emission wavelength of the NLuc-E2 nanoparticle may be red-shifted by using bioluminescence resonance energy transfer (BRET) pairs involving NLuc and fluorescent proteins, including mKate2 (λ_max_^EM^ ​= ​633 ​nm), CyOFP1 (Antares, λ_max_^EM^ ​= ​589 ​nm), and tdTomato (ReNL, λ_max_^EM^ ​= ​585 ​nm), or NLuc and fluorescent probes, including siliconrhodamine (λ_max_^EM^ ​= ​670 ​nm), carbopyronine (λ_max_^EM^ ​= ​645 ​nm) and tetramethylrhodamine (λ_max_^EM^ ​= ​585 ​nm) [16,[65], [66], [67]]. Furthermore, *in vivo* imaging of NLuc-E2 could potentially be improved by using alternative furimazine analogues, such as fluorofurimazine, for higher luminescence, sensitivity, and bioavailability [68]. Coelenterazine analogues have also been shown to shift the NLuc maximum emission to nearly 598 ​nm, although luminescent intensities can be significantly lower [69]. Besides NLuc, other luciferases for nanoparticle attachment for potentially better *in vivo* imaging include firefly luciferase, *Phrixothrix hirtus* railroad worm luciferase, and their mutants [16,70]; however, the significantly larger sizes of these systems [16,71] may result in lower luciferase-to-nanoparticle conjugation ratios.

NLuc or NLuc BRET systems have been used for *in vivo* imaging; however, the majority of the studies utilize NLuc-transfected cells for examining the progression of cancer growth or viral infection [15,72], and there are only a few examples of *in vivo* imaging using NLuc-labeled nanoparticles. Kamkawe *et al*. [73] demonstrated that NLuc conjugated on quantum dots (QD-Nluc) can be used for popliteal lymph node mapping and tumor-target imaging *in vivo*. Extracellular vesicles labeled with NLuc have also been shown for tracking their biodistribution [74,75]. Overall, the use of NLuc-labeled protein nanoparticles for *in vivo* imaging has not been extensively explored, and our studies here highlight advantages of using this type of system.

Our data suggest that nanoparticle-bound NLuc (e.g., NLuc-E2) has better signal-to-noise ratios and is a better imaging tool than conventional fluorescently-labeled nanoparticles (e.g., AF-E2) for *in vivo* and *ex vivo* imaging and for imaging that needs a lower detection limit. This supports observations that conventional fluorescent labeling of nanoparticles for *in vivo* tracking does not provide enough signal and sensitivity, which remains problematic [62,76,77]. As the results of NLuc-E2 *in vivo* and *ex vivo* studies are supported by our previous biodistribution work done with flow cytometry [44], NLuc-E2 can be utilized as an alternative platform for *in vivo* non-invasive imaging and *ex vivo* biodistribution studies with great sensitivity and fidelity, both being vital characteristics for imaging probes in biodistribution studies [75,78].

## 4 Conclusions

We implemented the SC-ST strategy for immobilizing bioluminescent proteins, NLuc and Akaluc, onto the exterior of the E2 nanoparticle for improved imaging. We demonstrated that immobilized NLuc can retain its bioluminescence at pH 5.0 with more than 2 orders of magnitude higher luminescence than free NLuc. Immobilizing Akaluc did not decrease luminescence at pH 7.4, suggesting that Akaluc-E2 can be utilized in red-shifted luminescence applications.

We examined the luminescence of NLuc-E2 and NLuc *in vitro* after cellular uptake and showed that NLuc-E2 persisted longer than NLuc. Furthermore, we were able to image NLuc-E2 after cell uptake with bioluminescence live-cell imaging. *In vivo* and *ex vivo* data showed the biodistribution of NLuc-E2 but not AF-E2 in the expected draining LNs up to 72 ​h after administration. Moreover, the biodistribution of E2 nanoparticles could be visualized with greater signal intensity and better signal-to-noise ratios using NLuc-E2 than AF-E2. As the interior of NLuc-E2 is available for conjugation of guest molecules, this versatile nanoparticle platform with multivalent NLuc displayed on the surface may be an attractive theranostic and bioluminescent scaffold for cellular, *in vivo*, and *ex vivo* imaging studies.

## Author contributions

**Enya Li**: Conceptualization, Methodology, Investigation, Data Curation, Formal Analysis, Visualization, Writing- Original Draft, Writing- Review & Editing. **Caroline K. Brennan**: Conceptualization, Methodology, Investigation, Formal Analysis, Visualization, Writing- Review & Editing. **Aaron Ramirez**: Methodology, Investigation, Writing- Review & Editing. **Jo Anne Tucker**: Investigation, Writing- Review & Editing. **Nina Butkovich**: Investigation, Writing- Review & Editing. **Vijaykumar S. Meli**: Investigation, Writing- Review & Editing. **Anastasia A. Ionkina**: Conceptualization**. Edward L. Nelson**: Conceptualization, Supervision, Writing- Review & Editing. **Jennifer A. Prescher**: Conceptualization, Supervision, Project Administration, Writing- Review & Editing**. Szu-Wen Wang**: Conceptualization, Supervision, Project Administration, Writing- Review & Editing, Funding Acquisition.

## Declaration of competing interest

The authors declare that they have no known competing financial interests or personal relationships that could have appeared to influence the work reported in this paper.

## Data Availability

Data will be made available on request.
