# Supplementary material for: Macromolecular assembly of bioluminescent protein nanoparticles for enhanced imaging
Source: Mater Today Bio. 2022 Oct 8;17:100455. doi: 10.1016/j.mtbio.2022.100455 (PMC9593766; doi:10.1016/j.mtbio.2022.100455)
Supplement: Multimedia component 1 [file mmc1.pdf]

Macromolecular Assembly of Bioluminescent Protein Nanoparticles for Enhanced Imaging

Enya Li[1], Caroline K. Brennan[2], Aaron Ramirez[1], Jo Anne Tucker[3],

Nina Butkovich[1], Vijaykumar S. Meli[1,4], Anastasia A. Ionkina[5], Edward L. Nelson[3,5,6],

Jennifer A. Prescher[2,5,6,7], Szu-Wen Wang[1,4,6,*]

[1] Department of Chemical & Biomolecular Engineering

[2] Department of Chemistry

[3] Department of Medicine

[4] Department of Biomedical Engineering

[5] Department of Molecular Biology & Biochemistry

[6] Chao Family Comprehensive Cancer Center

[7] Department of Pharmaceutical Sciences

University of California

Irvine, CA 92697, USA

Supplementary Information

Supplemental Methods

Conjugation of Maleimide Alexa Fluor molecules to the interior of E2 [1] and ST-E2

AF-E2: E2(D381C) was mixed with 8.5 molar equivalents of TCEP per E2(D381C) subunit for 1 h at room temperature to reduce the internal cysteines. 3 molar excess of AF750 per E2(D381C) was mixed with the reduced E2(D381C) for 2 h at room temperature, and 4 ºC overnight. Unreacted AF750 was removed with Zeba Spin Desalting Columns with 40K molecular weight cut-off (Thermo Scientifc) to get the final product (E2-AF750). The absorbance of E2-AF750 was measured at 753 nM with a plate reader (SpectraMax M2), and the conjugation ratio of E2(D381C) to AF750 was calculated based on a standard curve under the same buffer conditions.

AF568-NLuc-E2: The same protocol for conjugating AF750 was followed except ST-E2(D381C) was used. The next day after removing the unreacted AF568 from AF568-STE2, 0.7 to 1 molar ratio of SC-NLuc to ST-E2 subunit was mixed and left at room temperature for 2 h, then 4 ºC overnight.

Supplemental Figures

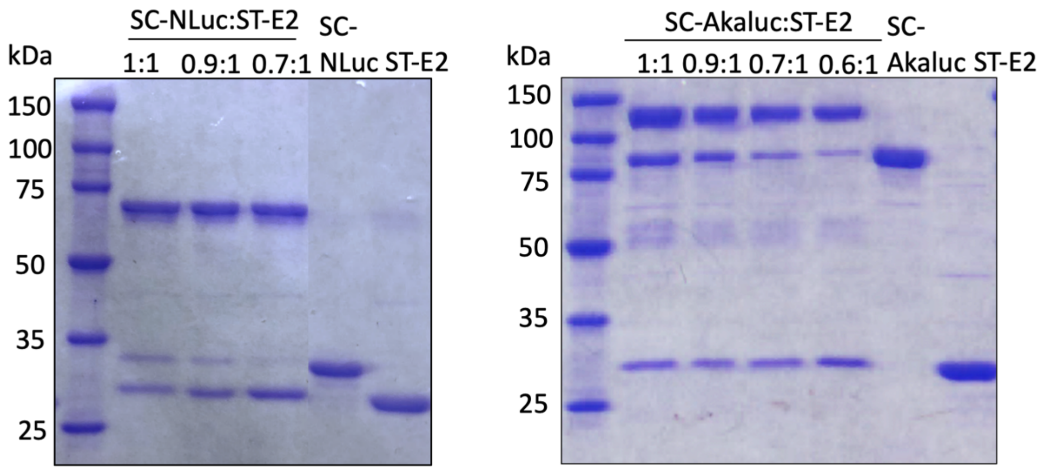

Figure SI-1. Different conjugation ratios of SC-bioluminescent proteins to ST-E2 affect the conjugation efficiency and the amount of unconjugated SC-bioluminescent proteins. The ratio shown above represents the ratio of SC-NLuc or SC-Akaluc to one ST-E2 subunit. To obtain the highest conjugation ratio of bioluminescent protein to E2 but minimizing unconjugated SC-bioluminescent proteins, we chose the molar conjugation ratios of NLuc and Akaluc to E2 monomer as 0.7:1 and 0.6:1, respectively.

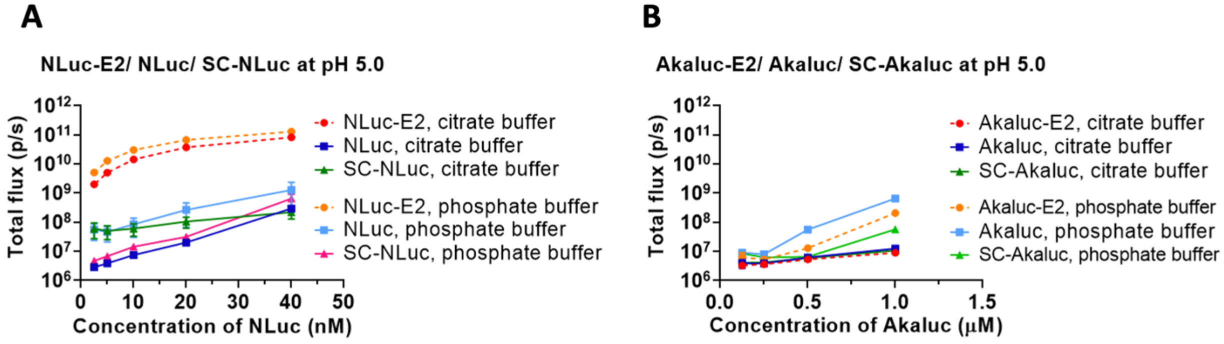

Figure SI-2. NLuc and Akaluc samples were diluted in pH 5.0 buffers to evaluate the enzyme performance under acidic conditions. (A) NLuc-E2, NLuc, or SC-NLuc (B) Akaluc-E2, Akaluc, or SC-Akaluc, was diluted in citrate buffer or phosphate buffer at pH 5.0. The luminescence for both NLuc and Akaluc groups was measured using IVIS. The total flux for each sample is plotted as mean ± SEM; n ≥ 3 for all groups.

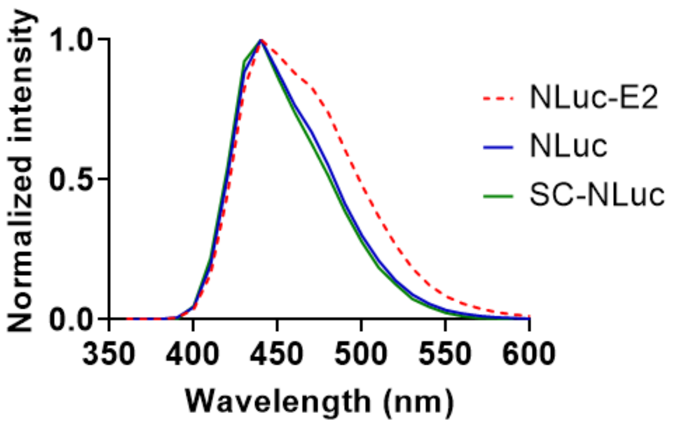

Figure SI-3. Emission spectrum of 15 nM NLuc-E2 (red dotted line), NLuc (blue line), and SC-NLuc (green line) from 360 nm to 600 nm. Intensity is normalized to the intensity at maximum emission wavelength after background subtraction from phosphate buffer. Each spectrum shown is an average measured from 3 independent samples.

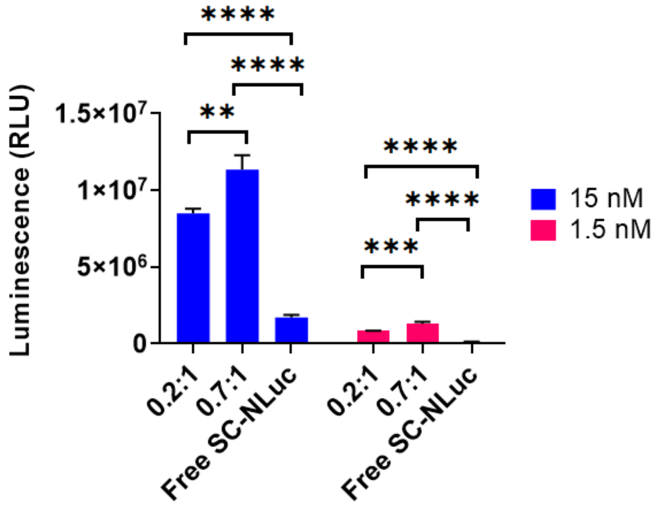

Figure SI-4. Effect of conjugation ratio on luminescence for NLuc-E2. SC-NLuc and ST-E2 were conjugated with a mixing molar ratio of 0.2:1 or 0.7:1 of NLuc to E2 subunit, and the luminescence was measured with the NLuc concentration held constant. An equal concentration of SC-NLuc served as a control. Values are shown as average ± SEM from 3 independent conjugations. Statistical differences were determined with one-way ANOVA with Tukey's multiple comparisons test (***p*<0.01, ****p* <0.001, *****p*<0.0001).

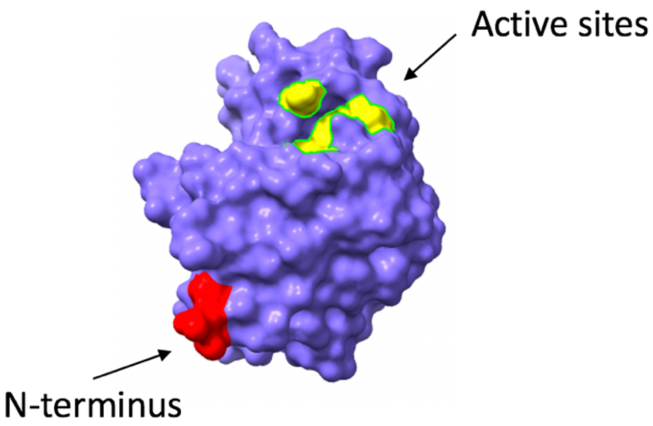

Figure SI-5. The structure of NLuc (in purple) was generated with ChimeraX using ID code from Protein Data Bank (5IBO) [2]. The N-terminus (in red) and active sites (in yellow) were colored using ChimeraX [3, 4]. By attaching SpyCatcher to the N-terminus of NLuc, the active site is on the opposite of the N-terminus and should be facing outwards when SC-NLuc is immobilized onto ST-E2.

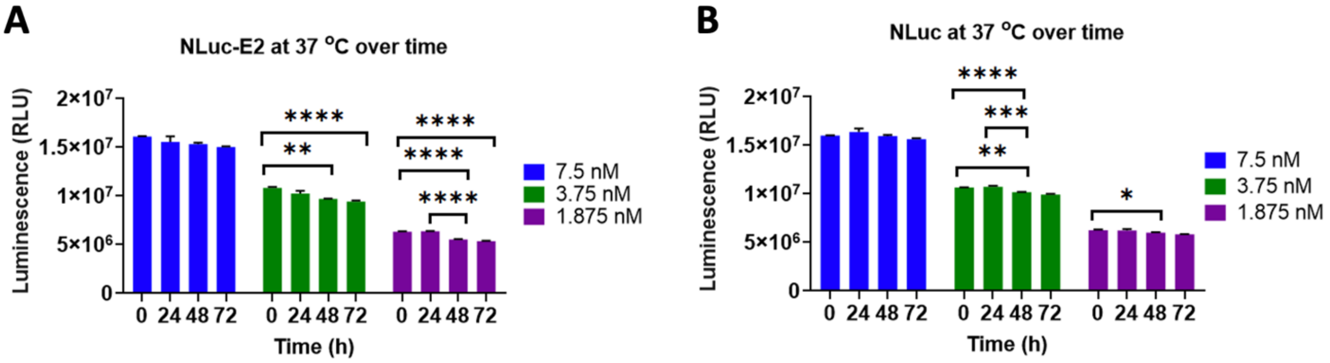

Figure SI-6. Thermal stability of NLuc-E2 and NLuc at 37 °C over time. (A) NLuc-E2 or (B) NLuc was serial diluted in media and placed in a 96-well clear bottom white plate and left at 37 °C incubator for 0, 24, 48, and 72 hours. The luminescence was read after to evaluate the stability of the samples. The concentration is represented as the concentration of NLuc in the NLuc-E2 or NLuc samples. The media background was measured and subtracted from all groups. Data are represented as average ± SEM; n = 3 for all NLuc-E2 and NLuc groups. One-way ANOVA with Tukey's multiple comparison test was performed to determine the significance (**p*<0.05, ***p*<0.01, ****p* <0.001, *****p*<0.0001).

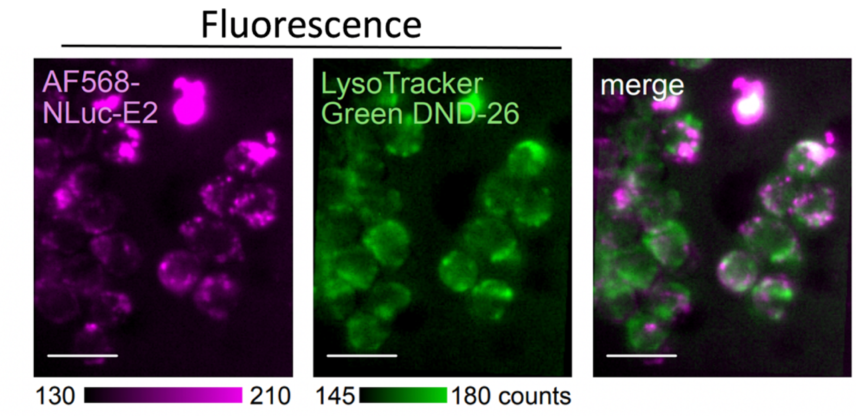

Figure SI-7. AF568-NLuc-E2 uptake by RAW 264.7 cells. After washing off excess AF568-NLuc-E2 after one hour of incubation of AF568-NLuc-E2 and RAW 264.7 cells, fluorescence from AF568-NLuc-E2 (magenta) and Lysotracker (green) were imaged. The fluorescent images were merged. Counts indicate intensity counts and scale bar = 25 μm.

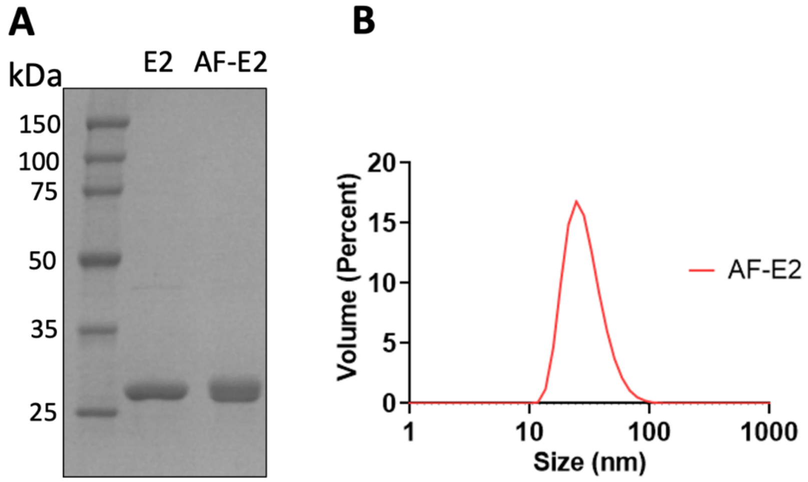

Figure SI-8. Characterization of Alexa Fluor 750 (AF) conjugated to the E2 nanoparticle (AF-E2). (A) SDS-PAGE of AF-E2 after conjugation. E2 alone was included on the gel as a control. (B) Hydrodynamic diameter of AF-E2 from three independent conjugations. AF-E2 shows a size of 29.7 ± 1.6 nm (n = 3).

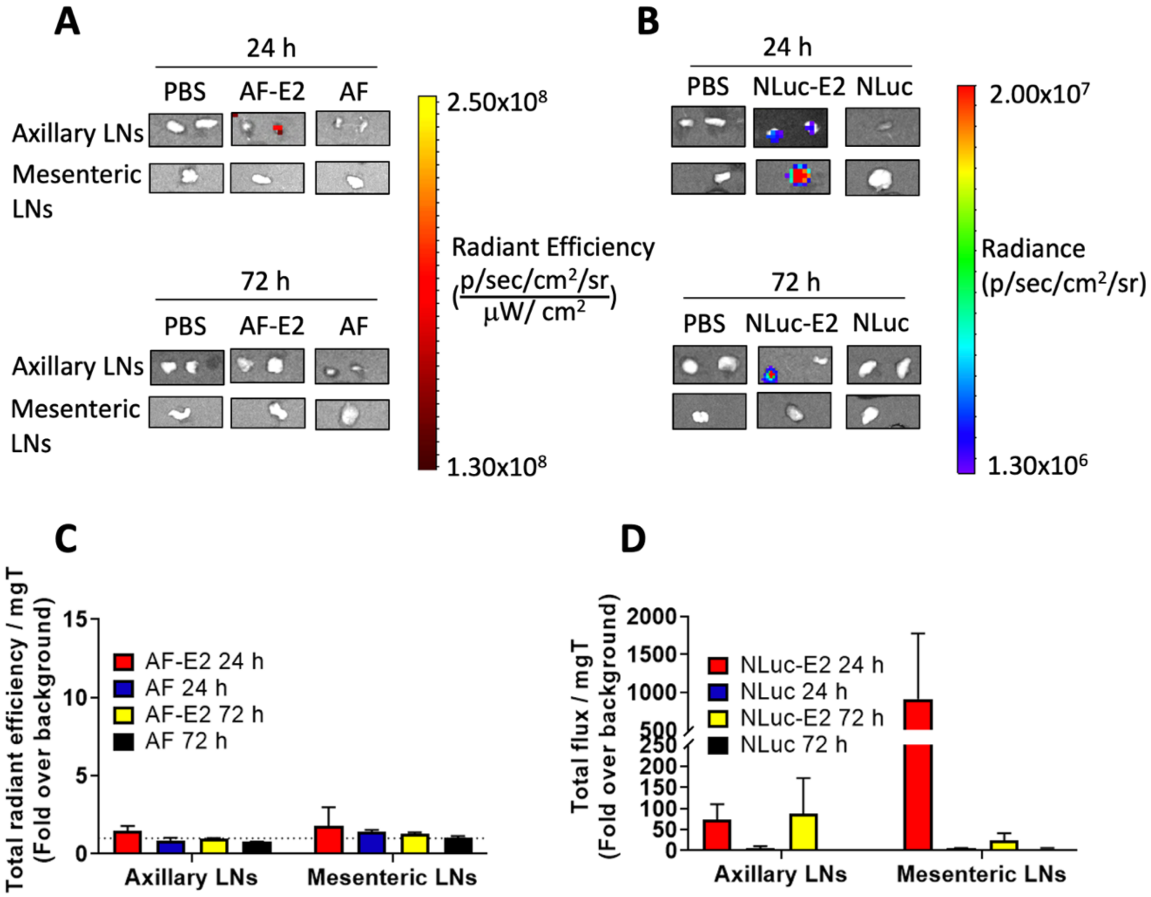

Figure SI-9. *Ex vivo* imaging and fluorescence and bioluminescence in axillary and mesenteric LNs. (A) Representative fluorescent and (B) bioluminescent images of the axillary and mesenteric LNs harvested at 24 and 72 h. (C) Fluorescence or (D) bioluminescence was quantified from the *ex vivo* images and normalized to its respective organ weight. Finally, data are represented as fold over its respective organs from the PBS-injected mice. The dotted line represents normalized PBS value at 1. Error bars represent standard error of mean; $n \geq 2$ for all groups.

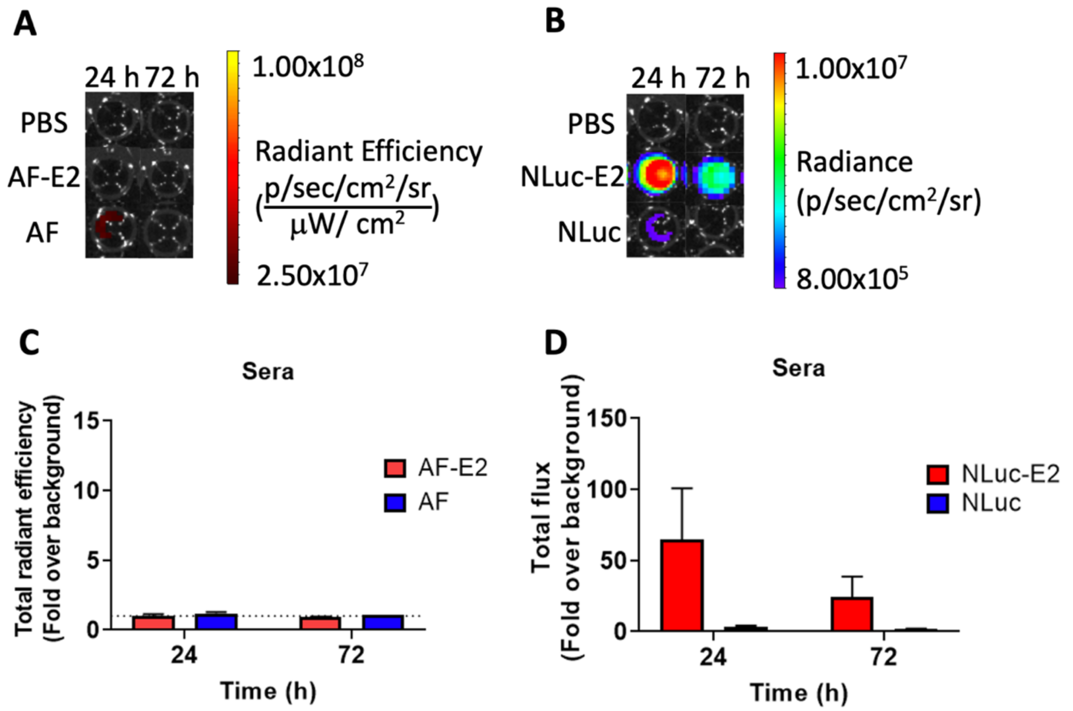

Figure SI-10. Fluorescence and bioluminescence in sera. (A) Representative fluorescent and (B) bioluminescent images of sera collected at 24 and 72 h. (C) Fluorescence from the sera of PBS, AF-E2, AF mice and (D) bioluminescence from the sera of NLuc-E2 and NLuc mice were measured. Data are represented as fold over the sera from the PBS mice. The dotted line represents normalized PBS value at 1. Error bars represent standard error of mean; n ≥ 2 for all groups.

Table SI-1. Primers used for cloning. All primers are named in the following order: restriction site, gene attached, and forward or reverse primer.

Primer name	Sequence
NdeI-SpyTag_Forward	CATATGGCCCACATCGTTATGGTGGATGCCTACAAGCCAACTAAAGGTTCAGGAACAGCAGGTGGTGGGTCAGGTTCCCTGTCTGTTCCTGGTCCCGC
BamHI-SpyTag_Reverse	GGATCCTTAAGCTTCCATCAGCAGCAGTTCCGG
NdeI-SpyCatcher_Forward	CATATGTCGTACTACCATCACCATCACCATCACG
NheI-SpyCatcher_Reverse	GCTAGCAATATGAGCGTCACCTTTAGTTGCTTTGCC
NheI-NLuc_Forward	GCTAGCGGTTCAGGAACAGCAGGTGGTGGGTCAGGTTCCGTCTTCACACTCGAAGATTTCGTTGGGG
BamHI-NLuc_Reverse	GGATCCTTACGCCAGAATGCGTTCGCACAG
NheI-Akaluc_Forward	GCTAGCGGTTCAGGAACAGCAGGTGGTGGGTCAGGTTCCGAAGATGCCAAAAACATTAAGAAGGGCCC
BamHI-Akaluc_Reverse	GGATCCTTACACGGCGATCTTGCCGTCC
NdeI-NLuc 6x HIS_Forward	CATATGGTCTTCACACTCGAAGATTTCG
BamHI-NLuc 6x HIS_Reverse	GGATCCTTACGCCAGAATGCGTTCGCACAG

Amino acids sequences used in this study:

SpyTag-Spacer-E2(D381C)
MAHIVMVDAYKPTKGSGTAGGGSGSLSVPGPAAAEEKAAPAAAKPATTEGEFPETREKMSGIRRAIAKAMVHSKHTAPHVTLMDEADVTKLVAHRKKFKAIAAEKGIKLTFLPYVVKALVSALREYPVLNTSIDDETEEIIQKHYYNIGIAADTDRGLLVPVIKHADRKPIFALAQEINELAEKARDGKLTPGEMKGASCTITNIGSAGGQWFTPVINHPEVAILGIGRIAEKPIVRCGEIVAAPMLALSLSFDHRMIDGATAQKALNHIKRLLSDPELLLMEA

6xHisTag-TEVsite-SpyCatcher-Linker-NLuc
MSYYHHHHHHDYDIPTTENLYFQGAMVDTLSGLSSEQGQSGDMTIEEDSATHIKFSKRDEDGKELAGATMELRDSSGKTISTWISDGQVKDFYLYPGKYTFVETAAPDGYEVATAITFTVNEQGQVTVNGKATKGDAHIGSGTAGGGSGS-
VFTLEDFVGDWRQTAGYNLDQVLEQGGVSSLFQNLGVSVTPIQRIVLSGENGLKIDIHVIIPYEGLSGDQMGQIEKIFKVVYPVDDHHFKVILHYGTLVIDGVTPNMIDYFGRPYEGIAVFDGKKITVTGTLWNGNKIIDERLINPDGSLLFRVTINGVTGWRLCERILA

6xHisTag-TEVsite-SpyCatcher-Linker-Akaluc
MSYYHHHHHHDYDIPTTENLYFQGAMVDTLSGLSSEQGQSGDMTIEEDSATHIKFSKRDEDGKELAGATMELRDSSGKTISTWISDGQVKDFYLYPGKYTFVETAAPDGYEVATAITFTVNEQGQVTVNGKATKGDAHIGSGTAGGGSGS-

EDAKNIKKGPAPFYPLEDGTAGEQLHKAMKRYALVPGAIAFTDAHIQVDVTYAEYFEMSVRLAE
AMRRYGLNTNHRIVVCSENSSQFFMPVLGALFIGVAVAPANDIYNERELLNSMGISQPTVVFVS
KKGLRKVLNVQKKLPIIRKIIIMDSKTDYQGFQSMYTFVTSHLPPSFNEYDFVPESFDRDKTIALI
MNSSGSTGLPKGVALPHRTACVRFSHARDPIFGYQNIPDTAILSVVPFHHGFGMFTTLGYLICG
FRVVLMYRFEEELFLRSLQDYKIQSALLVPTLFSCLAKSTLIDKYDLSSLREIASGGAPLSKEVGE
AVAKRFRLPGIRQGYGLTETTNAVMITPEGDRKPGSVGKVVPFFEAKVVDLVTGKTLGVNQRG
ELCVRGPMIMSGYVNNPEATNALIDKDGWLHSGDIAYWDEDEHFFIVDRLKSLIKYKGYQVAPA
ELEGILLQHPYIFDAGVAGLPDDDAGELPAAVVVLEHGKTMTEKEIVDYVASQVTTAKKLRGGV
VFVDEVPRGSTGKLDARKIREILTKAKKDGKIAV

6xHisTag-NLuc
MHHHHHHVFTLEDFVGDWRQTAGYNLDQVLEQGGVSSLFQNLGVSVTPIQRIVLSGENGLKID
IHVIIPYEGLSGDQMGQIEKIFKVVYPVDDHHFKVILHYGTLVIDGVTPNMIDYFGRPYEGIAVFD
GKKITVTGTLWNGNKIIDERLINPDGSLLFRVTINGVTGWRLCERILA

6xHisTag-TEVsite-Akaluc
MHHHHHHKLENLYFQMEDAKNIKKGPAPFYPLEDGTAGEQLHKAMKRYALVPGAIAFTDAHIQ
VDVTYAEYFEMSVRLAEAMRRYGLNTNHRIVVCSENSSQFFMPVLGALFIGVAVAPANDIYNER
ELLNSMGISQPTVVFVSKKGLRKVLNVQKKLPIIRKIIIMDSKTDYQGFQSMYTFVTSHLPPSFNE
YDFVPESFDRDKTIALIMNSSGSTGLPKGVALPHRTACVRFSHARDPIFGYQNIPDTAILSVVPF
HHGFGMFTTLGYLICGFRVVLMYRFEEELFLRSLQDYKIQSALLVPTLFSCLAKSTLIDKYDLSSL
REIASGGAPLSKEVGEAVAKRFRLPGIRQGYGLTETTNAVMITPEGDRKPGSVGKVVPFFEAKV
VDLVTGKTLGVNQRGELCVRGPMIMSGYVNNPEATNALIDKDGWLHSGDIAYWDEDEHFFIVD
RLKSLIKYKGYQVAPAELEGILLQHPYIFDAGVAGLPDDDAGELPAAVVVLEHGKTMTEKEIVDY
VASQVTTAKKLRGGVVFVDEVPRGSTGKLDARKIREILTKAKKDGKIAV

References:

[1] Dalmau, M.; Lim, S.; Wang, S.-W. Design of a pH-Dependent Molecular Switch in a Caged Protein Platform. Nano Letters 9 (**2009)** 160-166. DOI: 10.1021/nl8027069.

[2] Berman, H. M.; Westbrook, J.; Feng, Z.; Gilliland, G.; Bhat, T. N.; Weissig, H.; Shindyalov, I. N.; Bourne, P. E. The Protein Data Bank. Nucleic Acids Res 28 (**2000)** 235-242. DOI: 10.1093/nar/28.1.235.

[3] Altamash, T.; Ahmed, W.; Rasool, S.; Biswas, K. H. Intracellular Ionic Strength Sensing Using NanoLuc. International Journal of Molecular Sciences 22 (**2021)** 677. DOI: 10.3390/ijms22020677.

[4] Pettersen, E. F.; Goddard, T. D.; Huang, C. C.; Couch, G. S.; Greenblatt, D. M.; Meng, E. C.; Ferrin, T. E. UCSF Chimera – A Visualization System for Exploratory Research and Analysis. J Comput Chem 25 (**2004)** 1605-1612. DOI: 10.1002/jcc.20084.
